# Making “inclusion” more than a buzzword: A critical interpretive synthesis of literature about recruiting seldom-heard groups in health research

**DOI:** 10.1371/journal.pone.0318466

**Published:** 2025-06-12

**Authors:** Meghan Bradway, Lilisbeth Perestelo-Perez, Alezandra Torres-Castaño, Ana Maria Claudia Wagner, María Luisa Álvarez-Malé, Garlene Beatriz Zamora Zamorano, Kari Dyb

**Affiliations:** 1 Norwegian Centre for E-Health Research, University Hospital of North Norway, Tromsø, Norway; 2 Evaluation Unit, Canary Islands Health Service, Tenerife, Spain; 3 Endocrinology and Nutrition Department, Complejo Hospitalario Universitario Insular Materno-Infantil, Las Palmas de Gran Canaria, Spain; 4 Institute of Biomedical and Health Research (IUIBS), Universidad de Las Palmas de Gran Canaria, Las Palmas de Gran Canaria, Spain; 5 Department of Social Sciences, University of Tromsø - The Arctic University of Norway, Tromsø, Norway; University of Greenwich, UNITED KINGDOM OF GREAT BRITAIN AND NORTHERN IRELAND

## Abstract

**Introduction:**

Seldom-heard’ groups do not experience equitable access, engagement, or benefits from health services and are often underrepresented in research.

**Methods:**

This paper presents a critical interpretive synthesis (CIS) of the literature. The included articles were those published between 2000 and 2024 in PubMed and Web of Science that focused on the recruitment and engagement of ‘seldom-heard’, ‘hard-to-reach’, or ‘disadvantaged’ groups in Europe. Purposive samples of articles followed a flexible and iterative review. Analysis and synthesis focused on identifying “synthetic constructs” or overarching themes, which informed the development of a “synthesizing argument” regarding recruitment and engagement strategies for seldom-heard groups. The presented paper focuses on the “synthetic constructs”, whereas the “synthetic argument” will be described in a subsequent paper.

**Results:**

Of the 7601 articles identified, 23 were included. The analysis revealed four synthetic constructs: 1) the complexity of labels and identity, 2) the impact of labels on recruitment, 3) strategies to address recruitment challenges, and 4) the broader effects of these strategies on research and researchers.

**Conclusion:**

Effective recruitment of seldom heard groups requires lengthy, careful planning, relationship-building, and an understanding of their unique perspectives. In doing so, researchers can contribute to reducing health inequalities and amplifying the voices of underrepresented populations.

## Introduction

Recruitment of representative samples is a cornerstone of the feasibility and validity of any health study. The absence of participants prevents data collection, which leads to the absence of results and ultimately insufficient, ineffective or nonexistent knowledge production. Of course, this is an oversimplified argument, but the point is clear—health research must generate robust evidence on the effectiveness of interventions or concepts with relevant populations. Such evidence is essential to guide healthcare providers and policymakers in adopting the most feasible and effective health and care strategies for the public.

Unfortunately, participation rates are not ideal. The European Health Examination Survey Pilot Project (2009--2012) reported participation rates of 16--57% in men and 32--74% in women aged 25--64 across countries, despite the use of resource-intensive recruitment methods [1]. Factors influencing representativeness include recruitment methods, study design, and participant self-selection or nonparticipation [2]. Declining participation rates also vary by country and sociodemographic factors, further undermining the trustworthiness and generalizability of results [2]. The consequence is underreported—and therefore understood—of risk factors, such as health behaviors, in certain groups [3].

### Recruitment is a two-way street

Recruitment and retention, or engagement, of participants in health research studies require considerable motivation and effort from both the researchers and participants. However, significant barriers also arise on both sides of this dynamic process.

Historically, recruited samples have often failed to accurately represent the population, especially those considered seldom heard. The reasons for this are multifaceted, ranging from intentional to unintentional personal choices and research strategies that restrict the recruitment of certain groups from participating in health research studies [4,5]. This challenge has led to the adoption of terms such as “hard-to-reach” groups, and more recently referred to as “seldom-heard” groups [6]. The term “seldom-heard groups” is considered more appropriate, as it avoids implying “blame” or assigning sole responsibility to these groups because of their limited engagement with health research [6]. Other papers have described them as “under-represented communities, groups, populations or people who use or will potentially use services but who are less likely to be heard by professionals and decision-makers” [1]. However, there is no standardized definition of seldom heard groups. Acknowledgement of this diversity can be mapped and understood by considering the definitions presented by the authors of the articles, i.e., who the researchers themselves believed to be seldom heard or similar and why. This is also the intention behind our broad and inclusive search strategy described below.

Some reasons for the lack of participation from certain groups include self-selection (participants’ side) and exclusionary a-priori inclusion criteria (researchers’ side). Examples of self-selection are those with low digital literacy [7] or those with competing life priorities, such as family responsibilities or the immediate need to secure basic necessities [8]. These factors may eclipse researchers’ attempts to recruit participants, further complicating efforts to engage seldom-heard groups.

From the researchers’ perspective, inclusion and exclusion criteria are often designed to ensure that participants can provide the necessary data and that the study remains feasible. However, feasibility constraints, such as limited time and financial resources, may lead researchers to prioritize recruitment of more accessible populations, even when this compromises the representativeness of the sample [9]. A review of randomized controlled trials (RCTs) revealed an exclusion rate of 77.1%, with age and comorbidities being the most common exclusion criteria [10]. Such exclusions disproportionately impact marginalized populations, perpetuating inequities in health research outcomes [10]. These challenges are further exacerbated by long-standing systemic issues such as segregation, misconception and discrimination, which erode trust and communication between certain groups and health authority figures. These cross-generation scars, rooted in historical inequities, influence participation in health research even before engagement strategies are implemented [11].

### Purpose

Through the analytical framework of critical interpretive synthesis (CIS), this study aimed to examine and critically analyze the strategies employed by health researchers to identify, engage, and recruit seldom-heard groups. By developing a nuanced understanding of the factors influencing individuals’ relationships with their health and research, this study sought to identify challenges and facilitators of engagement and inform the development of more inclusive and representative approaches to health research. In doing so, we ensured their relevance and applicability to these underserved populations. The presented paper explores the first level of interpretation of the raw data—synthetic constructs—and presents the second level of interpretation—the synthesizing argument—as described by Dixon et al [12].

## Methods

### Design: Critical Interpretive Synthesis (CIS)

A CIS is a literature review methodology that adopts a qualitative, inductive approach to analyze and interpret the meaning embedded in the scientific literature [12]. Unlike traditional systematic reviews, CISs emphasize the synthesis of findings to generate new insights and theoretical understanding rather than merely summarizing existing evidence. Rooted in interpretative methods, the CIS aligns closely with meta-ethnographic approaches, which involve comparing and translating studies to identify overarching themes. However, the CIS is distinguished by its capacity to incorporate and synthesize diverse methodological approaches, including qualitative, quantitative, and mixed-methods studies [13–15]. The main characteristics of a CIS review are:

- The use of a “compass” research question [16], subject to adjustment based on the findings in the literature,
- Flexible inclusion/ exclusion criteria based on relevance to the compass question [12]
- Conducting additional interpretations and synthesis on identified themes

The literature search and analysis adhered to the methodological framework outlined by Dixon-Woods et al [12] and followed the reporting criteria proposed by Depraetere et al [17] in addition to Preferred Reporting Items for Systematic Reviews and Meta-Analyses (PRISMA) checklist [18] (S1 File). The review process consisted of a purposive sampling approach to identify relevant peer reviewed literature, followed by primary and flexible inclusion criteria to narrow the scope to answer the research question within a collection of comparable studies, a reassessment of previously excluded articles that appeared relevant, and finally analysis of selected articles via inductive thematic analysis and interpretive synthesis to identify and analyze connections between themes.

In lieu of a formal protocol, detailed descriptions of the data extraction, analysis, and synthesis processes are provided in S2 File, including examples of the analytical steps and mapping processes used to derive synthetic constructs.

### Literature search

This CIS of the literature explored how researchers identified, reached out to, and engaged those described as ‘seldom heard’ in primary health research. We applied a purposive and broad search strategy by identifying articles published between 2000 and 2024 in PubMed/Medline and Web of Science. The first search was performed on January 22, 2023 for articles published between the years 2000–2022 and an updated search on May 16, 2024 for articles published between the years 2022–2024, with the overlap in dates ensuring that no relevant literature was excluded for the period. Search terms followed the PICO search strategy:

- Population: seldom-heard, “hard to reach”, disadvantaged, underserved or unengaged adults (population)
- Intervention: recruitment and engagement strategies for health research studies
- Comparison: not applicable
- Outcomes: efficacy of recruitment and engagement strategies for this target group

The scope was limited to studies conducted in the World Health Organization’s European Region [19], that involved adults identified as part of a ‘seldom-heard’ (or similar) group, as defined by the authors of each article. The full search string is available in S2 File.

### Literature selection

Titles and abstracts were screened for inclusion using Rayyan, an online system to facilitate systematic reviews [20]. After duplicates were removed, we identified titles and abstracts of articles that explicitly or potentially described: 1) adults who are considered seldom-heard groups, 2) definitions for “seldom heard” groups, 3) targeted recruitment of these groups and 3) focused inquiry or intervention on primary health prevention. Articles were excluded if they 1) involved stakeholders other than seldom-heard groups, 2) were performed outside of the EU, 3) reported outcomes solely on the outcomes of a program or research materials, e.g., standardized questionnaire, development or cost-effectiveness and 4) described seldom heard groups as those related to vulnerability toward a health condition. We also excluded articles that intentionally focused on COVID. However, those whose outcomes included unintended results related to COVID were considered for inclusion. The full list and description of inclusion and exclusion reasons are included in S2 File.

A group of preliminary reviewers (1st reviewer and two research assistants) reviewed titles and abstracts concerning topics outside the scope of this review: wrong publication types (protocols, reviews), wrong study design (ones in which recruitment of the target group was not performed), not primary prevention (focused on a pre-existing condition), etc. The first reviewer (MB) conducted all rounds of review, supported by iterative ‘pilot reviews’ of 20-50 randomly selected abstracts with all members of the interdisciplinary research team to refine and scope of the compass question. Uncertainties were marked and later discussed amongst all reviewers. In line with the CIS approach, complementary and associated articles were also considered. Both groups of articles were those that did not initially meet inclusion or exclusion criteria but were relevant to the compass question [12]. The 1st reviewer reviewed all included full-texts for reference to other relevant articles, websites, registrations or reports (e.g., trial registration databases) about the study described. These are considered “associated articles” and serve to support the depth of understanding of the researchers’ experiences and positions. Complementary articles were those that were originally excluded but ultimately included based on relevance to the research question.

### Quality assessment

The goal of this review was not to evaluate the success of the studies but to provide an overview of the perceptions, motivations and strategies used by researchers during recruitment and retention of seldom heard groups. Therefore, quality was based on the depth and quality of information regarding how participants were considered, treated and approached for inclusion in the studies. This allowed us to focus efforts on understanding what Edwards et al described as the “signal” (evidence that is relevant to our research questions), as opposed to the “noise” (methodological quality) [12,21].

Therefore, quality was predicated on three principles. The first was the Declaration of Helsinki’s definition of ethical research and reporting practices for health studies involving human subjects. Basic ethical requirements were: 1) provision of adequate study information to potential participants to form the basis of informed consent, 2) the collection of informed consent prior to study start by those capable of providing it voluntarily, or an appropriate proxy, 3) a description of receiving ethical approval or exemption (with justification), from an credible institution or ethical committee, and 4) that participants’ privacy was respected via the anonymization or de-identification and secure storage of collected data [22]. The second was the targeted and sole recruitment of those who were identified as ‘seldom-heard’, which would ensure that recruitment and engagement strategies identified in the text were specific to our target group. Previously excluded yet relevant articles were included regardless of this because these were often secondary research and may not have these details. The third was the exclusion of articles that were deemed “fatally flawed”, i.e., studies that involve “ethical, paradigmatic, theoretical or methodological issue”. Examples presented by Glenn Richey and Davis‐Sramek that were particularly useful included: data collection methods that could not provide evidence enough to answer research questions – or there was a lack of research question or objective-, a lack of positioning or theoretical basis, the purpose or relevance of a study in its given field (academic contribution) were not well described or justified, or unbalanced and biased perspectives such as naïve positivism [23].

### Data extraction

Raw data were extracted verbatim from the articles and systematically organized using Excel to minimize interpretive bias during the extraction phase. Data extraction included: *Study details*: Title, authors, date of publication, and country/region of study. *Study design and focus*: Research design, health focus, and target groups. *Target group characteristics*: Reasons provided for classifying groups as seldom heard. *Recruitment strategies*: Locations, personnel involved, and materials used. *Enrollment methods*: Ethical approval, informed consent, and inclusion/exclusion criteria. *Participation outcomes*: Recruitment goals, enrollment numbers, measures of follow-up and attrition, and any compensation provided.

### Analysis and synthesis

The iterative analytical process followed Dixon-Woods et al’s framework [12] (S2 File). This allowed us to explore new questions that emerged during the analysis, consistent with the dynamic nature of CIS. The analysis combined inductive thematic analysis with interpretive synthesis, focusing on identifying thematic connections and synthesizing insights across studies.

Groups of tables compared raw data by emergent themes, which enabled the development of what is described by Dixon-Woods et al as “synthetic constructs” [12]. These served as a foundation for mapping relationships and patterns between themes and topics and exploring their implications in greater detail. These tables included: target group and all descriptions of target group (which were subsequently categorized for easier review); personnel (and their role in recruitment), recruitment material; recruitment location, formal method, criteria; factors of retention and attrition; recruitment goals and enrollment numbers. Relationships between topics or themes were then mapped and subsequent questions were asked. Articles were reviewed again for any data that could clarify these questions (see S3 File). The development of a “synthesizing argument,” or a higher-order interpretation, integrates the “constructs” into a preliminary theoretical framework [12]. This will be the focus of a subsequent publication.

## Results

**Fig 1:**
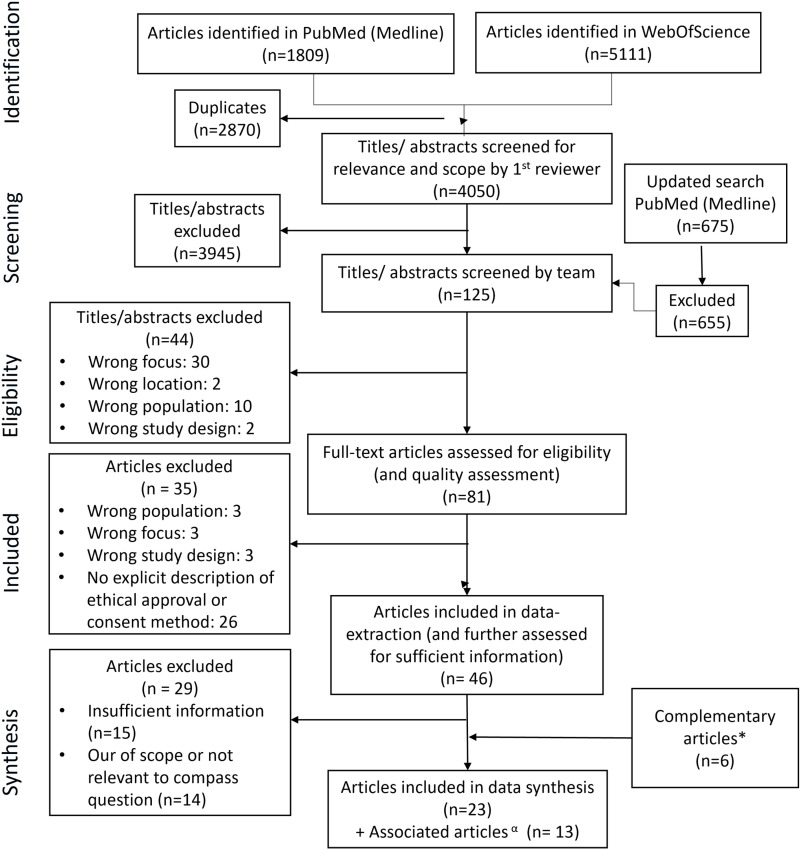
PRISMA diagram adapted for the CIS. * Complementary articles: articles that do not engage target group members directly. Instead, they describe relevant experiences reported by researchers or other stakeholders in the recruitment and engagement of target group members in health studies. α Associated articles: provide additional information on included articles, e.g., protocols and other reported results. For ease of reporting and readability of the manuscript, only the original included articles will be referenced in the manuscript. These results present the “synthetic constructs” of the CIS, focusing on first level interpretations of strategies for recruitment and engagement used to identify, reach and engage seldom heard groups in studies about primary health. These are all playfully summarized by familiar rock and pop songs (Fig 2).

The first literature search resulted in n = 6920, with n = 675 articles in the second. Due to the broad search criteria, it was necessary to perform two rounds of title and abstract reviews. After removing duplicates (n = 2870), the first round excluded articles that were blatantly out of scope or unrelated to the topic (n = 3945). The second round excluded n = 44 articles based on team discussions that clarified and focused the scope of relevant literature. After quality assessment and inclusion of relevant, but previously excluded, articles (n = 6), n = 23 articles were included in data synthesis (Fig 1).

**Fig 2:**
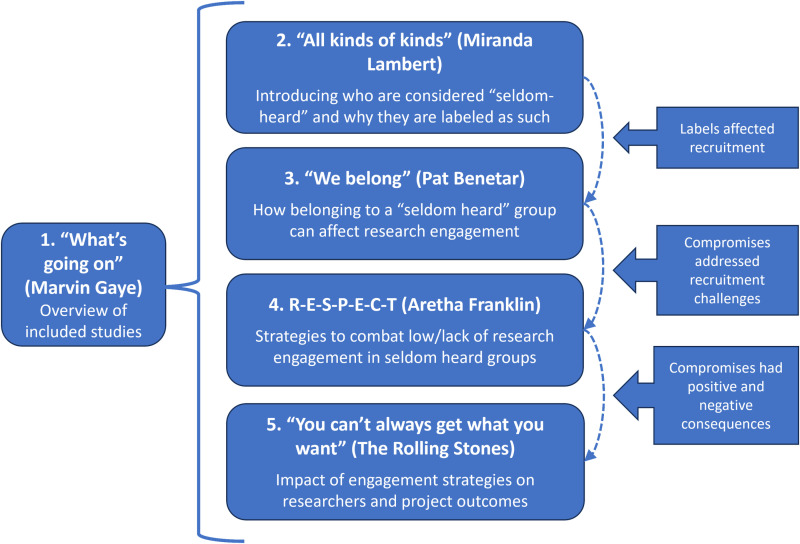
Illustration of how the results are organized (numbered) and related to one another (right) in a process of engaging seldom-heard groups.

### 1. “What’s going on?” (Marvin Gaye)

The circumstances surrounding a person’s life should affect our expectations of their involvement and contribution to research studies. As such, study designs are representative of what and how much effort are expected from target group members to participate. Table 1 is intended to provide this contextual information. Most studies were performed in Scotland or UK (n = 11), followed by two each in Germany and Ireland and one each performed in Sweden, Switzerland, Belgium, Poland the Netherlands and Denmark. Health promotion, prevention and wellness (n = 8) [24–31] were the most common health conditions described, followed by alcohol or other substance use (n = 5) [32–36], mental health (n = 3) [37–39], cancer (n = 2) [40,41], physical activity (n = 2) [42,43], heart disease (n = 1) [44], obesity (n = 1) [45], and primary healthcare use (n = 1) [46]. Terms used to describe target groups were assigned by authors and/or associated literature. Articles described or defined target group members as seldom heard according to one or more of the following: their gender (n = 8) [24–28,32,33,42], social or economic disadvantage (n = 8) [28,32,33,35,40,42,43,45], geography (n = 7) [28,34,36,37,40,43,45], participation in an existing program (n = 6) [24–28,38,42], age (n = 3) [32,33,40], occupation (n = 3) [34,37,38]), immigration status (n = 2) [26,27], and reception of social aid (n = 1) [36].

**Table 1:** Descriptions of target groups and their recruitment to studies related to primary health.

Authors [reference]	Location	Study design	Intervention	Health condition	Target group
**Kreiml, V., et al.** [42,47]^*^	Germany	Interviews	The BIG project	Physical activity	Socially disadvantaged women participating in BIG classes
**Mcgrath, A., et al.** [24,25,48]^*^	Ireland	Implementation-cost effectiveness [24], 10 week intervention [25]	Sheds for Life	Health promotion and prevention	Men’s Sheds members
**Irvine, L., et al.** [32]	Scotland/ UK	Intervention group from a larger RCT	[Text and multimedia messages]	Reducing alcohol consumption	Socially disadvantaged young to middle aged men
**Crombie, I.K., et al.** [33,49,50]^*^	Scotland/ UK	Parallel-group, pragmatic, individually randomized controlled trial	Texting to Reduce Alcohol Misuse	Reducing alcohol consumption	Socially disadvantaged young to middle-aged men
**King, E., et al.** [37]	Scotland	RCT, Interviews	Living Life to the Full for Farmers	Mental health	Farmers and agricultural workers, individuals from the farming community, individuals who encounter people from the farming community
**Lock, K., et al.** [34]	UK	Longitudinal, qualitative panel study	[Smoke-free legislation]	Smoking cessation	Workers in North London
**Lindsjö, C., et al.** [26,27,51]^*^	Sweden	Story-dialog method	Collaborative Innovations for Health Promotion	Health promotion	Women migrants context from the co-creative lab
**Thielecke, J., et al.** [38,52]^*^	Germany	Semi-structured interviews with participants from a larger RCT	Recruited form the project ‘With us in balance’	Serious mental health	Farmers from a personalized telephone coaching program
**Helitzer, E., H. Moss, and J. O’Donoghue** [28]	Ireland	Mixed methods (survey, focus group)	[Community Choir]	Health promotion	Women from disadvantaged area participating in a Community Choir
**Mcrobbie, H., et al.** [45,53,54]^*^	UK	Parallel group randomized controlled trial	Multi-modal task-based group intervention (Weight Action Program)	Obesity	Residents of economically deprived boroughs
**Kolovou, V., et al.** [40,55]^*^	UK	RCT	Awareness and Beliefs About Cancer (abacus3)	Cancer	Residents of socioeconomically deprived areas
**Grazioli, V.S., et al.** [35]	Switzerland	Questionnaires	Harm-reduction drop-in center	Substance use	Socially marginalized alcohol and other drugs users
**D’Hooghe, S., et al.** [43,56]^*^	Belgium	Walk-along interviews and focus group	CIVISANO-project	Physical activity	socioeconomically disadvantaged persons from peri-urban areas
**Milcarz, M., et al.** [36,57–59]^*^	Poland	Cross-sectional study	PL-13 Program, “Reducing Social Inequalities in Health”	Reducing tobacco use	Social assistance beneficiaries from Piotrkowska district
**Sinclair, A. And H. Alexander** [44]	Scotland	Interviews	The Keep Well initiative	Coronary heart disease	People who failed to attend a health check
**Craddock, E.** [29]^α^	UK	Semi-structured interviews	Women’s Health Network	Health and wellbeing	Members of the Women’s Health Network (WHN)
**Mueller, J., et al.** [41]^α^	UK	Semi-structured interviews	“Let’s be clear, get it checked!’	Cancer	Those involved in intervention management and delivery
**Bodewes, A.J. and A.E. Kunst** [46]^α^	Netherlands	Primary: Analysis of recruitment strategies Secondary: low budget surveys	[Low budget health surveys]	Healthcare use	Moluccans
**Ridley, J., S. Hunter, and A. Rosengard** [39]^α^	Scotland	Focus groups and in-depth interviews	The Mental Health (Care & Treatment) (Scotland) Act 2003 implemented in 2005	Mental health	Mental health carers (experiencing mental health challenges)
**Bysted, S., et al.** [30]^α^	Denmark	Recruitment strategies	[Staffed intervention provided by municipality]	Health promotion	Residents from disadvantaged neighborhoods, adults with mental, social or physical issues
**Lewis, S., et al.** [31]^α^	UK	Qualitative data (1^st^ phase) of the Communities in Control study	Big Local *(ongoing)*	Public health decision making	Residents from disadvantaged neighborhoods

Data availability: See S2 File for article selection, S3 File 1 for study details, Table 2 for verbatim group descriptions, and Table 3 for recruitment and engagement strategies.

* Associated articles that provide additional information on included articles, e.g., protocols and other reported results. For ease of reporting and readability of the manuscript, only the original included articles will be referenced in the manuscript.

α Complementary literature: articles that do not engage target group members directly. Instead, they describe relevant experiences reported by researchers or other stakeholders in the recruitment and engagement of target group members in health studies.

More detailed descriptions are included in S3 File.

### 2. “All kinds of kinds” (Miranda Lambert)

Before exploring how seldom-heard groups were recruited and engaged in health research, we first explored how authors described them. The most commonly used terms were “marginalized”, “disadvantaged”, and “deprived”. However, some authors noted that these terms should be taken with a grain of salt; they are labels assigned by those who exist in a world or reality outside that of the target groups – identities that are often not shared by target group members. This is consistent with another topic mentioned by Lindsjö, et al [27], i.e., situated knowledge in which knowledge generation is described as tied to a person’s social situation and identity [60]. In a study of social and emotional wellbeing in an all-female community choir, the term “regeneration area” was seen as derogatory and stigmatizing by choir members. In fact, several participants insisted that “we’re not a disadvantaged area. We’re a community” [28].

The authors suggested that the nonhomogeny of a group, i.e., the diversity within a group, renders typical analysis ineffective. Not only should we be aware of subgroups, but we should also question the limitations or scope of who is considered a “member” of a labeled group [43]. One article argued that their definition of “who is relevant to asking about understanding smoking risk” was more inclusive than others; all social aid beneficiaries who had less accurate knowledge of smoking risk, which included but was not limited to current tobacco smokers [36]. Acknowledging the “multiplicity of individuals and groups”, i.e., subcultures of subgroups, and its relationship with motivation is necessary for participation [31].

The relationship between the diversity of a group and intersectionality, or multiple discrimination, compounds the complexity of one’s identity. In walk-along interviews, participants walked researchers through their neighborhoods, pointing out factors that limited their capacity to engage in regular walking [43]. A follow-up focus group categorized the reasons they were considered “persons in socioeconomically disadvantaged situations (PSEDS)” and “less likely to engage in recreational walking”: stigmatization, sociocultural environment, financial barriers, information availability and personal literacy [43]. Authors urged future research to use an intersectional approach to comprehensively understand health inequalities experienced by seldom heard groups.

### 3. “We belong” (Pat Benetar)

Here, we explain that a group’s context (self-perceived identity, externally placed labels and diversity) impacts strategies used to identify, recruit and engage those groups. As described above, culture and belonging are common factors in assigned labels or self-assigned identities of groups which affect subsequent behaviors and how they are treated in society, healthcare and research.

#### Culture and belonging.

“Culture” is notoriously hard to define and measure with respect to health [61]. While cultures can be differentiated, in this paper we acknowledge the broader concept of culture as shared features which are not mutually exclusive. People can belong to multiple cultures, which can be factors for or against engagement in research [61].

Culture was identified in included articles as common health beliefs, e.g., “if I don’t know about something, I cannot worry about it” [40]; gender norms, including men avoiding seeking mental and physical health care for fear of appearing weak, emasculating or stigmatized [24,25,37]; immigration experience that impacts access to resources and presence at common research recruitment venues [27], or residing in a “regeneration area” that is subsequently stigmatized [28], etc.

Health promotion and interventions often take place in communities, which involve diverse individuals who share such things as social interactions, locations and beliefs, of which people also experience differently [62]. Communities are also tied to culture where people have a “shared language, geography, habits and customs”, tradition, common beliefs, etc. [63].

Social connections between community members are the foundation for the snowballing recruitment method, which can increase the reach of recruitment and participation [40,43,45]. Peer support was also referred to as social cohesion by Lindsjö et al [26,27]. Similarly, the social learning effect [45], was described as motivating, engaging and offering support, thereby establishing a sense of belonging [26–28,45].

Shared emotions, e.g., fear, also contribute to social cohesion and community. For members of a women’s community choir group, fear of societal repercussions from “them” (i.e., those who marginalize them owing to their residence in a deprived area) was their motivation for coming together. Ironically, they also believed that because of their strong comradery, “they” were not encouraged to participate [28].

Personal values and cultural history can affect ethnic or societal cultures and subsequently lead to self-exclusion. For example, Moluccans valued privacy surrounding the topic of personal health, which often led to (self-)exclusion in health studies. Moluccans were also part of a culture with a generational history of mistreatment by the Dutch government [46], which could have influenced their value of independence. Previous personal experiences of disappointment with research could also affect willingness or openness toward research participation [36].

Occupation can also serve as the basis for community and culture. In two studies, farmers and seasonal workers noted a common cultural belief that having mental health challenges was a character flaw and that seeking help for one’s mental health was emasculating [37]. Low help-seeking and research recruitment challenges were also associated with their inability to take holidays, uncertain weather conditions, seasonal work leading to high mobility, and other factors outside of their control [37,38].

#### Participatory design.

Belonging was also used as a tool to engage target groups in research. Researchers took advantage of the potential to learn from other perspectives through participatory approaches, including a variety of stakeholders, [27]. Collaborating groups were valued for their expertise and insights, often engaged representatives of seldom heard groups and made space for their voices to be heard in a research context.

External partners contributed to planning by serving as representatives [24–27,29,37,41,42], advocacy groups or local social services [26,27,36,37,39,40,43], financial or insurance institutions [24,25,38], individual healthcare providers or representative organizations [24,25,30,32,33,37,40,44,45], and existing community programs [24–29,31,35,38,42,46].

During the planning phases, these groups facilitated the development of research strategies [24–27,34,37,38,41,42]. During recruitment, external partners provided lists of eligible participants from their member databases [33,36,43] and informed and screened potential participants (via digital, in-person or analog methods). Examples include in-person meetings with existing community programs or healthcare facilities [24,25,42,44] and letters of invitation at common locations, or on their websites [30,33,35,38,40,41,43,46]. In two related “no-contact” studies, general practitioners served as a buffer between researchers and participants by delivering the recruitment materials based on target group preferences [32,33].

During studies, partners facilitated participant retention and engagement by reinforcing trust in, and credibility of, the study [26,27,30,38,41,46]. Some trained lay health advisors (described below) [26,27] or delivered intervention components themselves including health checks [24,25,30], phone-based helplines [37], on-site coaching or other socioeconomic services [38] or speeches during intervention meetings. Others served as sites for data collection via surveys [43], clinical visits [45], and interviews or focus groups [43,44]. In some studies, the existing community program was the studied intervention and therefore had already established and were successful at reaching and engaging target groups [24–28,30,31,35,42].

Lay health advisors were a specific type of partner who represented both the research team and target group simultaneously. This built a bridge of communication and provided more in-depth and contextualized understanding of target groups [26,27,40,41]. Because participants were comfortable with and related to these individuals, lay health advisors were able to facilitate recruitment, data collection and interpretation.

Some members of the target groups (not lay health advisors) were also representatives within the research team, in addition to participating. These individuals contributed to protocols, engagement strategies and materials [40]. They were considered key recruitment personnel [46], especially during snowballing recruitment [43] or respondent driven sampling [32], and served as validators of research findings [28,38,43]. However, one study cautioned against high expectations of such individuals as partners; there is a limit to their capacity to contribute, e.g., that they are not familiar with the health vernacular, are not professionals and/or already experience multifaceted burdens in daily life [29].

### 4. “R-E-S-P-E-C-T, Find out what it means to me!” (Aretha Franklin)

In this section, we describe how and why participatory design facilitated researchers’ respect for target groups. According to Lysaught, respect is “to regard her or him highly—to esteem, honor, value in his or her uniqueness or distinctiveness, to make space for the person to be him- or herself” [64]. In other words, there is a need in research practice to expand our perception of a group prior to requesting any level of engagement from them. However, the challenge to do so was owed to the challenges of balancing respect for participants with the implications and duties of a study [28].

In the included articles, researchers frequently noted the importance of giving relevant groups space and a voice, building trust during this process of engagement and autonomy, as either the original intent or emergent outcome of their studies. While it is expected, especially for seldom-heard groups, that participant engagement dwindles as the study progresses, we were surprised that most described their activities as effective at reaching participants and maintaining engagement [24–28,32,34,38,40].

#### Knowledge leads to active acknowledgement.

Respect was demonstrated by acknowledging situations and contexts in which target groups live, and anticipating their needs before trying to approach or recruit them. External partners contributed insights into personal, practical and societal aspects of target groups that could impact their engagement in their health and health research.

Research teams tailored recruitment and intervention expectations to the personal needs of their target groups, e.g., during recruitment, lay advisors collected preferred methods and follow-up times [40]. A drop-in center for harm reduction (not abstinence) let “clients” consume alcohol and drugs at the center, in a safe environment. Counseling was always present for clients but was never a requirement [35]. Tailored outcome measures focused on discretion and realistic expectations [35]. In a study of (former) non-attenders of health checks, it became apparent that what works for one may not work for others. In fact, some participants reported that they needed to be pushed lightly with telephone calls or even home visits to eventually visit a health checker whereby these same methods would be a deterrent for others [44]. Program activities of a long-standing program for promoting physical activity amongst disadvantaged women were based on a cooperative planning group in which women in the target group were primary decision-makers [42]. Settings for activities centered around respecting the needs of all-women groups from a range of cultural backgrounds, e.g., closing the blinds during interviews with women with Muslim faith [42]. Then, of course, there is the tried-and-true engagement factor – compensation [33,38,40,43,45,46].

As described above, language reflects culture. Some research groups tailored data collection approaches to the language of their target groups. In the co-creation lab program for health, the inclusion of lay health advisors was reportedly meant to act as an interpreter of the language as well as cultural meaning behind responses [26,27]. In the two studies of a no-contact text-message drinking intervention, the vernacular of disadvantaged men in Scotland was key to engagement [32,33]. In another study, a steering group member of a cancer awareness program mentioned that tailoring the language of the intervention – or methods of using engaging and friendly materials that reflect social culture – of the intervention was what made the intervention successful [41].

History was understood and acknowledged on personal, cultural and generational levels. For migrant women from the co-creative lab in Sweden, a personal history of poor self-esteem, negative beliefs about the future and lack of social support were brought upon by social discrimination based on their ethnicity, social status and gender. As such, the development of the program focused on addressing the resulting isolation, lack of access to, and priority of, health and care options [26,27]. One researcher, with Moluccan ancestry, attempted to acknowledge the Moluccan’s history of mistreatment during tailored recruitment. Unfortunately, these attempts were not enough to overcome such a history of abuse; survey responses rates within the districts ranged from 9% to 58% [46].

Researchers also acknowledged practical limitations – most commonly time – when tailoring activities to lower the barriers to participation. For example, health promotion activities were based on availability of target group members [26,27]. In another study, time and capacity constraints led researchers to decrease interview times and offer the opportunity to schedule an interview immediately after (eventually attended) typical health checks to increase convenience. Even so, approximately 30% of those approached still reported having too little time to participate. Other reasons were that family responsibilities took priority over their own health, and that the discomfort one felt when requesting time away from work to attend a health check [44].

#### From trust, to autonomy, to ownership.

Many studies specifically attributed their success with recruitment and retention to trust building. Trust allowed researcher to promote target groups as equals, or experts, of their unique situations through autonomy in and ownership of interventions. It was also credited for contributing to the successful engagement of seldom-heard groups [26,27,31].

Some studies reported repeatedly visiting the recruitment site prior to recruitment to build relationships to promote familiarity and comfort [24–27,33,40]. Collaborating with existing community programs provided a sense of credibility and trustworthiness of researchers [24–28,31,35,42]. In the study of a co-creative lab program, researchers incorporated an explicit “trust-building phase” [26,27]. Most reported the research team, lay health advisors, community program staff or healthcare providers performing in-person recruitment or intervention delivery for the expressed purpose of building trust [24–27,33,40,42]. In-person efforts had several benefits; establishing rapport, facilitating participant engagement, and maximizing reach of recruitment efforts [24,25,30,40,43], as well as establishing the perception of trustworthy information [41], promoting comfort [26,27], and demonstrating respect for culture [28,40,46].

Researchers empowered participants through support and education and included them in the decision-making process. In one study, authors described the participants as “experts” which justified the participants’ choice of route during their walking interviews, which allowed researchers to be educated about which challenges, factors and environments were relevant to this group [43]. In the co-creative lab, health circle meetings included in the intervention were specifically meant to “to reduce power construction and as a symbol that all the women were equal”. In fact, all community members discussed the topic of culture and power in focus groups on a regular schedule. These factors resulted in reports that the intervention was “advantageous for health knowledge acquisition”, that the women could learn by doing, and that the setting provided a space for women to be heard. Participants reported that this empowered them to consider speaking up and participating in community decision-making related to health.

Researchers supported community ownership and autonomy by training lay health advisors to take over the intervention delivery and sustain the benefits of the intervention after the study officially ended [26,27]. In the study of the Big Local program, researchers invited community members to participate in public health policy by putting them in positions of power where they could directly impact decisions. In fact, autonomy was considered an “ethical imperative” [31].

Ownership, as the result of facilitating autonomy and empowerment in several studies, was described as key to intervention sustainability. Previously described studies have promoted concepts such as “collective control” [31], development of career and involvement in the initiative [26,27], and emphasizing ownership by allowing participants to determine their own level of participation [24,25].

#### Pitfalls of participatory design: exclusion.

While most reported successful inclusion through extensive recruitment and retention efforts, some argue that the idealized participatory design principles are often not reached in earnest. Instead, attempts are often considered tokenistic, or partners and participants are not truly representative of the target group. In the included studies, barriers to achieving a representative sample stemmed from both the research teams and participants perspectives.

However, this was not for lack of trying. Unintentional exclusion was described by authors as the result of the unknown cultural value of privacy in relation to health topics [46]. Even lay health advisors, as target group members, could not completely avoid the problem of unintentional exclusion when they relied on the limited scope of their own social networks for recruitment [26,27]. Physical barriers also limited reach and engagement of specific groups who were unaware of flyers posted at inaccessible parts of the city [31]. Participation barriers occurred when researchers had not acknowledged a group’s identity, including non-homogeny [43] or differences between sub-cultures [25,26,28,31,37].

While not common in these studies, inclusion criteria are intentional reasons that can lead to non-representativeness. One study required that “key informants…be approached easily” [46], which is the opposite of the term hard-to-reach. Another study required participants to “be able to distance from suicidal ideation” – a mental health state that would benefit from such coaching [38]. The target groups described in other studies were described as ethnic minorities and economically and socially deprived, yet exclusion required ability to read, write or understand English [24,25,40,45], or being registered with a GP, which may be difficult for disadvantaged groups [45].

However, researchers in these cases also took measures to specifically ensure that participants were representative of the target groups in other ways, e.g., only including those who were officially defined, via the Welsh Index of Multiple Deprivation, as economically deprived [40], or accepting any co-morbidity “the study addressed the needs of the National Health Service (NHS) and the results are generalizable to target populations” [45].

Regardless of how much effort is put into bringing research to the participants, there is still the potential for them to refuse to participate. Such self-selection can be caused by disinterest or perceived lack of relevance [40]. Formal impediments, i.e., ways of speaking, communicating and vernacular used in more formal arenas can also discourage participation [46]. Another study argued that the exclusion of “lay lay” members, as the result of a traditional hierarchy of expertise or knowledge of lay users over lay-lay users, was to blame [29].

This does not mean that efforts are fruitless. Craddock argued that even though those participating in the network were not representative, they can still act as conduits – bridging communication between those whom they represent and healthcare providers [29].

### 5. “You can’t always get what you want” (The Rolling Stones)

In this section, we present the positive and negative consequences of the tailored recruitment and engagement strategies.

First, the positive consequences. By defining recruitment and intervention activities based on proximity to target groups, studies were able to reach participants who may experience one of the most common barriers – distance from a service or resources [30]. As previously mentioned, these included normal workflow of an existing health setting [30,44], areas people frequent [33,40,41,46] and natural smoking settings [34]. However, Lewis et al reinforced the risk of choosing a specific location; what may be accessible for one part of the target group, may be further away or more difficult to attend for others [31].

In one study, interviews with participants revealed that the promotion of the equality and physical space of the intervention was a major motivational theme and facilitator of health learning [26,27]. This was attributed to the physical and mental space outside of the home or work – also called “third places” – in which the participants could retreat and find a sense of familiarity and safety from discrimination [26,27]. Other groups valued distance which allowed for visual anonymity and the option to participate from a familiar setting [38]. In another study, researchers emphasized that the no-contact properties of text messaging met the needs of young and middle-aged disadvantaged men in Scotland who showed reluctance toward direct contact [32].

However, most studies used in-person strategies which minimized not only the physical but also inter-personal distances between researchers and members of target groups. For example, the employment of lay health advisors, or target group peers, to present information in a familiar setting contributed to building trust, establishing credibility among the community while also enhancing cancer awareness among residents [41]. Healthcare providers reported that performing house-to-house recruitment increased familiarity (putting a face to the service and making it more tangible) and shortens the path between thinking about and actually using the services [30].

Now for the downsides. By acknowledging the challenges that seldom groups face, prior to study start, the researcher could tailor recruitment and engagement strategies (S3 File Table 5). However, our finding was that often what made it easier for participants, made it “harder” for researchers. For example, forming relationships with partners, building trust and understanding of the needs of target groups was time consuming and resource demanding for research team members [26,27,30,37,40]– as were efforts to promote convenient, safe and inclusive atmospheres for participants [26,27,30,32,37,45]. For example, flexible participation strategies led to drop outs and inconsistent or unreliable data [24,25,27,31,42,45], and lengthy planning and tailoring of recruitment activities to the culture and history of groups, such as the Moluccans, led to delays and dwindling financial resources [46].

## Discussion

### The power of “doing our research” before doing our research

This CIS review effectively provided a story or explanation of the strategies used, and associated factors affecting the reach, recruitment and engagement of seldom-heard groups in health research. This emphasizes that health research does not occur in a vacuum. We, as health researchers, must acknowledge that we are imposing our agenda on groups who may not have the necessary resources and circumstances to engage. The authors of the included articles demonstrate the importance of “doing our research” before doing our research. They showed that respect and a willingness to adjust their practice to those whom they aim to help is effective in the engagement of seldom heard groups.

There seemed to be clear and related conceptual and practical steps that research teams used to target seldom heard groups: participatory design, learning about the group prior to their participation, tailoring recruitment and engagement strategies, building trust and empowering target group members. Briefly, by including external and community-based organizations during planning phases, researchers took the time to learn about the personal, cultural, practical and historical perspectives that could then inform the tailoring of research strategies. Two concepts – credibility and trust- seemed to extend beyond the delivery of recruitment materials and permeated the foundation of study activities. Taking the time to build trust between the researchers and target group members was an investment in the “success” of recruitment and engagement [26,27,31]. Strategies included: investing time in building relationships with target group members prior to study start, increasing transparency and availability of research team members, promoting comfort by tailoring study settings to target group preferences, providing resources and information to which they previously lacked access, empowering them to take control of their own health decision making, promoting a more equal power balance between participants and stakeholders and researchers [24–27], and even promoting collective control of the community to determine resource allocation [31]. These strategies were found to promote sustainability of the intervention or program even after study completion [26,27].

### Power differentiation: hierarchy in research

There is an obvious, yet rarely discussed, difference in power – a hierarchy – between researchers and researched. Understanding the power hierarchy is more commonly discussed about patients and healthcare providers; researchers and organizations, such as partners, researchers and healthcare providers; etc.; and not researchers and individuals [65]. This hierarchy in research manifests as practices that – unintentionally or intentionally – limit who participates in health studies. The authors and researchers involved in included articles successfully identified, reached and engaged seldom heard groups because they actively combated the causes of power differentials between researchers and researched. This is in line with the findings of Malla, Aylward and Ward, who describe the role between the power of knowledge, or lack there of, and perpetuating health inequalities [66]. They argue that power is given – from funding, political agenda and cultural variables, and even the biased labels of “relevance”, power is given to certain types of research and researchers and is restricted to others [66]. Just as those who as considered “seldom heard”, research in certain areas is limited by resources leading to what is referred to as “research imperialism”, i.e., research being driven by external agendas, not the needs of the public [67]. This sheds light on some of the up-stream and sometimes biased influences that affect who and what research focuses upon. This will be the focus of a follow-up paper but is described in part below.

### Adapting research strategies to the needs of seldom-heard groups

The recruitment of seldom heard groups challenged many assumptions and conventions of traditional health research, which emphasize objectivity, strict protocols, replicability of studies, and statistical significance [68,69]. In fact, a main trend in the recruitment and engagement of seldom heard groups was a decrease in distance both physically and metaphorically – between researchers and participants, researchers and partners, and social communities and scientific research communities. Pragmatic designs were also common; researchers argued that flexibility and adaptability were necessary to address uncertainties, e.g., target groups or settings as sporadic or inconsistent [24,25], mobile [37], hidden [36], untrusting or cautious toward outsiders [26,27,36,44,46].

### Fostering empowerment

The World Health Organization defines empowerment as a process through which “patients understand their role, are given the knowledge and skills by their health-care provider to perform a task in an environment that recognizes community and cultural differences and encourages patient participation***”*** [70]. In other words, it takes the acquisition and use of knowledge, resources and opportunity to enact one’s will. The process of empowering individuals, meaning making them aware of their own power – knowledge of their knowledge – e.g., situational knowledge and resources that can enable them to have greater agency over their own situation. However, this is easier for those who already have the capacity, knowledge, and resources to rise to the occasion of engaging with their health and the healthcare system [71]. Others – those without access or resources to build knowledge or those experiencing societal structures that repress their power – need to be given these things or enabled to enact their level of power by creating a new structure based upon their current capacity and agency [72].

Several studies provided learning opportunities and materials and support to target group members that they may not have had access to previously. This led to increased autonomy and, in some cases, ownership and sustainability of interventions or their teachings. This is reflective of the cyclical relationship of empowerment, i.e., self-determination or self-efficacy and patient activation [73,74]. In fact, many reviewed studies followed the factors and interactions mentioned in the proposed Patient-Centered Multi-Level Personalized Patient Activation and Empowerment Framework [74] which describes system-level support, i.e., involvement of multiple stakeholders required to facilitate participants’ knowledge and confidence when making health decisions.

This review also highlighted that while learning about and tailoring research to target groups created extra work and a longer study process, researchers reported that specific efforts to make participation more convenient and inclusive were worthwhile.

### Strengths and limitations

#### Strengths.

The CIS method was confirmed to be an appropriate methodology as we noticed a strong connection between themes or topics. The CIS allowed us to better describe how researcher intentions, identities of seldom heard groups, and strategies for targeted recruitment and engagement of these groups were related. Understanding the needs and capacities of seldom heard groups requires a more subjective stance, critical and qualitative look at what is presented in a paper, acknowledging the multisectoral complexity of factors. It is during the iterative stages of interpretation that our interdisciplinary team held an advantage by providing a comprehensive interpretation of what a group of articles indicates about a situation. As such, the CIS provides an avenue through which more evidence can be used to generate actionable knowledge in health research that is useful for healthcare providers, fellow researchers, policy makers, individuals and other stakeholders.

#### Limitations.

We acknowledge the limitations of only including two literature databases for this review. This was based on the arguments made by Dixon Woods et al that the purpose of a CIS is not meant to be exhaustive or aggregative, like systematic reviews, but instead to provide an overview of a situation to initiate the development of theory [12]. Also, critical appraisal tools for even the most standardized of literature reviews, such as the Critical Appraisal Skills Program (CASP) checklist for systematic reviews with meta-analysis of observational studies, only consider whether the review used a minimum of two databases to search for relevant literature [75]. We also limited the scope of health concerns to primary health concerns. This choice was based upon the parent project which aims to develop an AI driven app to aid individuals with primary health and prevention. Additionally, by limiting the scope, we ensured that studies, researcher perceptions and target groups’ situations were comparable, which would aid in the development of theory.

## Conclusion

We must understand that our assumptions, expectations and labels (historical) generationally limit both the effectiveness of research to provide knowledge on seldom heard groups. Current exclusion in research may present the image of “low valued people”. As such, it justifies people’s feeling of not being seen, which fuels the divide in terms of knowledge production, understanding, cooperation and potential benefit.

To call themselves “inclusive”, studies and community interventions must employ broad inclusion criteria and limited exclusion criteria (to an extent). Some expectations might be accurate, e.g., that it will take more effort and energy to address these groups, some assumptions of people’s willingness, or interest and value in participating could be entirely false. However, if all projects were to account for or address at least some of the factual and real limitations on both ends of the researcher-researched spectrum, we could increase representativeness of outcomes and impact for those who are currently seldom-heard. We intend to present the synthesizing argument of this CIS review [65] in a subsequent paper.

## Supporting information

S1 File Preferred Reporting Items for Systematic Reviews and Meta-analyses (PRISMA) Checklist. (DOCX)

S2 File Literature review and analysis protocol. (DOCX)

S3 File Mapping of data for synthetic construct development. (DOCX)

## Abbreviations

CIS: Critical Interpretive Synthesis
NA: Not available
RCT: Randomized control trial
