# Supplementary material for: Making “inclusion” more than a buzzword: A critical interpretive synthesis of literature about recruiting seldom-heard groups in health research
Source: PLoS One. 2025 Jun 12;20(6):e0318466. doi: 10.1371/journal.pone.0318466 (PMC12161589; doi:10.1371/journal.pone.0318466)
Supplement: S2 File — (DOCX) [file pone.0318466.s002.docx]

# **S2.** Literature review and analysis protocol

Here we describe the article review process, divided into rounds based on iterative discussions amongst all authors. While it is difficult to explicitly describe each decision made to determine eligibility or relevance of articles and their associated studies, we aim to be as transparent as possible.

CIS approach

We follow the recommended six main tenants of a critical interpretive synthesis presented by Depraetere et al [1]:

1. Open research or “compass” question which can be refined during the process of review
2. The literature search should be broad and accompany a structured approach
3. The literature selection should focus on relevance and be flexible
4. The quality appraisal should be based on relevance and “theoretical contribution to the CIS” and ideally answer the “Appraisal prompts for informing judgements about quality of papers” proposed by Dixon et al [2]
5. The data extraction requires reflexivity (authors self determination of bias in interpretation)
6. The formulation of a synthesizing argument (which will be presented in a subsequent paper) focuses on identifying the relationships between concepts presented in the literature with the aim of forming a “coherent theoretical framework”. [1]

## Full search strategy

Databased searched included PubMed (Medline) and Web of Science for the following terms in the title or abstract:

Population: “hard to reach” OR “difficult to reach” OR hidden OR underserved OR disadvantag* OR marginaliz* OR unengag* AND (population OR group OR communit*) NOT (adolescent OR child OR children OR infant OR youth)

AND [Field]: health OR self-manag*

AND [Study]: intervention OR survey OR study OR trial

AND [Focus]: recruit* OR participa*

NOT [Article types]: “literature review” OR “systematic review” OR “scoping review” OR “review of the literature” OR “narrative review” OR “this review” OR protocol OR retrospective*

Publication date: 2000-2022 (*an updated search was performed in 2024 for additional literature published between 2022-2024)

NOT [in title only]: provider* OR nurse* OR student* OR professional* OR staff OR clinician* OR stakeholder* OR physician* OR GP

## Screening

**Round 1.** Screening for and exclusion based on titles and abstracts

A group of preliminary reviewers (1^st^ reviewer and two research assistants) reviewed titles and abstracts concerning the topics and labels included in Table 1.

**Table 1.** Exclusion and label used to denote exclusion during each round of review

| **Label** | **Reason** |
| --- | --- |
| **Emergent group** | - assumed that recruitment activities did not specifically target this group   - not presented as seldom heard or similar in introduction, i.e.   - identified as seldom heard and why in results or conclusion   - they state that they are looking for associations that would indicate seldom heard/underserved populations (meaning they are not specifically sought after in recruitment) |
| **Secondary research** | - studies that used population data or previously collected data by others to explore a hypothesis |
| **Wrong publication type** | - Protocol - Review - Incomplete results |
| **Wrong location** | - Studies performed in any country outside of the UK or Europe - Exclude even if the study includes the UK or a country within Europe |
| **Not primary prevention** | - Describes prevention of the symptoms or worsening of a currently diagnosed health condition - **Note: If someone is described as having one health condition but the study is about prevention of a separate health condition, then the article should be included* |
| **Not health** | - About any field outside of human health care, intervention or self-management |
| **Wrong population** | - Healthcare professionals - anyone <18years old - stakeholders (not specified as individuals within the target group) - others included as participants: participants not defined within the target group, even if those from the target group were also included - students of health-related fields about their potential jobs in healthcare (**note: may include if these are groups described as seldom-heard etc. in primary health research or care*) - Carers |
| **Wrong study design** | - Case studies - Development of method tested on target group, e.g. new standardized questionnaire |
| **Wrong year** | - Before 2000 |
| **Wrong focus** | - Reporting of the outcomes of a program, e.g. cost-effectiveness, attendance rate without mention recruitment of individuals in target group. Those that include recruitment details will be included. - COVID mentioned as a focus of the study - “Vulnerability” label related to vulnerability to health condition, not healthcare service access or research participation |

*Note: when any reviewers were uncertain, abstracts were marked with why they were unclear, and these continued through the rounds of abstract and full-text until a conclusive answer was identified.*

**Round 2.** Clarifying the scope

Based upon the labels and questions that resulted from Round 1, a random sample of 50 of the remaining abstracts was presented and discussed amongst all authors as examples of the type of abstracts that were present to further clarify the scope.

Remaining titles and abstracts were excluded if they: did not mention search terms (our connotation of the search terms, e.g. not disparities or disadvantages in health outcomes, vulnerable in terms of progress of health condition, not determinant of health), described the wrong population (perceptions of or experiences by healthcare providers or other stakeholders, not about or related to individuals in the target group), performed the “wrong” study design (case study, studies only involving one person, no description of target group participation), performed secondary analysis (describes use of data collected by another group or study), included the wrong population (had to recruit only those from the target group, i.e. so as to ensure that recruitment protocols were tailored to the target group and not others), or had incomplete results (ongoing studies, preliminary findings).

**Round 3.** Assessing eligibility based on the presence of essential information needed to address the compass question

Iterative pilot rounds of abstract reviews including all reviewers (i.e. all authors and research assistants) to ensure presence of information about target group, i.e. description of, or indication that there is potential for, the following information in the full-text:

- why author(s) consider this group to be seldom heard
- who author(s) consider to be seldom heard
- The sole target of recruitment was the seldom heard target group

**Round 4.** Assessing eligibility based on further discussion of scope amongst all reviewers

An updated search was performed for articles between 2022-2024. All abstracts were tagged with at least one label – regardless of exclusion or inclusion - to aid in discussions amongst the authors of the scope. Iterative reviews of 20-50 abstracts were conducted, including all reviewers.

The scope of “primary health” and “prevention” were discussed amongst the research team at length and ultimately agreed upon. The following concepts were deemed “out of scope” for the purposes of this paper:

1. Should the focus of “health” such as cessation of tobacco use, smoking, drug and alcohol consumption or treatment of a primary condition be considered as primary health prevention?
   - Articles were excluded if participants were diagnosed with a condition, e.g. addiction, and the intervention focused on treatment of that condition.
   - Articles were included if target group members were described as seeking care without a formal diagnoses or label.
2. Was “access to general and/or primary health care” considered primary prevention? No, because primary health care can include treatment of existing conditions. It is too broad.
3. If articles were relevant for what could be considered background information they were marked with a label of which information could be relevant for future review, e.g. secondary research, reporting a researcher’s own experiences, program staff experiences etc.

**Round 5.** Assessment of eligibility based on Full text review

Abstracts that had been previously excluded because they were 1) secondary research or 2) researchers’ reflections about experiences with the target groups, without primary recruitment of target groups, were reviewed for inclusion. They were included if they presented the following details:

- recruitment materials and/or information,
- method of recruitment (does not have to be a formal description, e.g. purposive recruitment), AND
- who was involved in recruitment OR location of recruitment OR duration of recruitment period OR inclusion/exclusion criteria

**Round 6.** Associated documents reviewed for relevant information

The 1^st^ reviewer reviewed all included full-texts for reference to other relevant articles, websites, registrations or reports (e.g. trial registration databases) about the study described. These were assessed for the same criteria as Round 5 and data extraction would consider data from all “associated” documents or websites with information about the study. This was especially important if an included full-text article did not describe a key piece of information within that text, but referred to another source of that information.

Round 8. Quality Assessment

Quality assessment was based on a tailored list of requirements generated by the reviewers. The 1^st^ reviewer ensured that the necessary ethical considerations were taken, i.e. description that ethical approval was described and that it was granted and of the informed consent process and collection of signature or verbal consent to participate. Previously excluded articles that were later included in Round 6 were exempt as they may not have included primary recruitment of the target group.

## Data extraction

A preliminary data extraction plan included: title, authors, date of publication, country(ies) where studies were performed, study design, health focus, target groups, reasons for being considered target groups, study duration, recruitment strategy (location(s), personnel and materials), enrollment methods (ethical approval and informed consent), inclusion/exclusion criteria, goal for recruitment, achieved enrollment number, follow-up and repeated measures, non-participation and/or attrition, compensation.

Raw data, i.e. the text, was copied and pasted verbatim from the articles into an excel sheet, so as to ensure authenticity of descriptions by limiting interpretation of the text prior to analysis. Data extraction, analysis and synthesis stages occurred iteratively and cyclically (see below).

## Analysis and Synthesis

Iterative groups of tables comparing topics were made for easier analysis and development of synthetic constructs including: target group and all descriptions of target group (which were subsequently categorized for easier review); personnel (role in recruitment), recruitment material; recruitment location, formal method, criteria; factors of retention and attrition; recruitment goals and enrolled. Relationships between topics or themes were then mapped and subsequent questions were asked.

An example of synthetic construct development is in relation to questions about “partners” is described here: who was involved in recruitment and what were their roles? Patterns then emerged about the roles including proximity to target group members, i.e. direct contact with participants, consulting as partners to the research team when planning recruitment strategies or both? This led to consideration of who partners were: were they part of the community, organizations, healthcare professionals, target group members themselves? What was the relationship between who partners were, recruitment activities and location of activities? On that same note – how did members of the research team interact with participants?

## References

1. Depraetere, J., et al., *The critical interpretive synthesis: an assessment of reporting practices.* International Journal of Social Research Methodology, 2021. **24**(6): p. 669-689.

2. Dixon-Woods, M., et al., *Conducting a critical interpretive synthesis of the literature on access to healthcare by vulnerable groups.* BMC medical research methodology, 2006. **6**: p. 1-13.
