# Supplementary material for: Making “inclusion” more than a buzzword: A critical interpretive synthesis of literature about recruiting seldom-heard groups in health research
Source: PLoS One. 2025 Jun 12;20(6):e0318466. doi: 10.1371/journal.pone.0318466 (PMC12161589; doi:10.1371/journal.pone.0318466)
Supplement: S3 File — (DOCX) [file pone.0318466.s003.docx]

# **S3.** Mapping of data for synthetic construct development

**Table 1.** Summary description of included articles in critical interpretive synthesis

| **Authors [reference]** | **Location** | **Study design** | **Intervention** | **Health condition** | **Target group** | **Participant flow**  *[Goal, Approached, Screened, Baseline, Follow up N]* |
| --- | --- | --- | --- | --- | --- | --- |
| **Kreiml, V., et al. [1]** | Germany | Interviews | The BIG project, one-time interviews with long-term participants (at least 1 year in BIG classes) | Physical activity | Socially disadvantaged women participating in BIG classes | - **Goal:** NA - **Baseline:** n=30 |
| **Mcgrath, A., et al. [2, 3]** | Ireland | Implementation-cost effectiveness [2] and  10 week intervention [3] | Sheds for Life 10-week intervention, data collection over -3, 0,3 (following 10 week intervention),6,12 months | Health promotion and prevention | Men's Sheds members | - **Goal:** n=44 She centers, n=565 active members - **Baseline:**  n=31 Shed centers, n=421 Shedders - **Follow up 1:** n=421 Shedders (n=379 intervention, n=87 control) |
| **Irvine, L., et al.[4]** | Scotland | Intervention group from a larger RCT | NA | Reducing alcohol consumption | Socially disadvantaged young to middle aged men who are seldom in contact with health services | - **Goal:** NA - **Baseline:** n=67 (n=34 intervention (reported in this paper), n=33 control) - **Follow up 1:** 82% responded to text message question, 613/646 text messages delivered - **Follow up 2:** 19/27 text messages that did not require a response, were responded to |
| **Crombie, I.K., et al. [5]** | UK | Parallel-group, pragmatic, individually randomized controlled trial | Texting to Reduce Alcohol Misuse (TRAM) | Reducing alcohol consumption | Socially disadvantaged young to middle-aged, disadvantaged men | - **Goal:** n=20 GP practices, n=20 venues, n=798 individuals (n=319 + 20% attrition per group) - **Screened:** n=3603 - **Baseline:** n=825 (n=411 intervention, n=414 control) - **Follow up 1**: n=366 intervention, n=371 control - **Follow up 2:** n=349 intervention, n=358 control |
| **King, E., et al. [6]** | Scotland | Interviews | “Living Life to the Full for Farmers” (LLTTFF) | Mental health | Farmers and agricultural workers, individuals from the farming community, individuals who encounter people from the farming community in Scotland | - **Goal:** NA - **Baseline:** n=21 |
| **Lock, K., et al. [7]** | UK | Longitudinal, qualitative panel study | NA | Smoking cessation | Workers living or working in an area of North London | - **Goal:** NA - **Baseline:** n=32 - **Follow up 1:** 23 + 10 additional were successfully recruited to replace data from those lost to follow up |
| **Lindsjö, C., et al. [8, 9]** | Sweden | Story-dialogue method | Collaborative Innovations for Health Promotion | Health promotion | Women migrants in a Swedish context from the co-creative lab | - **Goal:** NA - **Baseline:**  n=21 [8] - **Goal:** NA - **Baseline:**  n=17 [9] |
| **Thielecke, J., et al. [10]** | Germany | Semi-structured interviews with participants from a larger RCT | Recruited form the project ‘With us in balance’ | Serious mental health | Farmers who took part in a personalized telephone coaching as part of the ‘With us in balance’ project | - **Goal:** n=20 - **Approached:** n=66 - **Baseline:** n=20 - **Follow up 1**: n=17 |
| **Helitzer, E., H. Moss, and J. O’Donoghue [11]** | Ireland | Mixed methods (survey, focus group) | NA | Health promotion | Women from an area of disadvantage | - **Goal:** n=6-12, - **Approached:** n=16 current members - **Baseline:** n=9 |
| **Mcrobbie, H., et al. [12]** | UK | Parallel group randomized controlled trial | Multi-modal task-based group intervention (Weight Action Program) | Obesity | Residents of economically deprived boroughs in London | - **Goal:** n=330 (n=109 control, n=221 intervention), - **Approached:** n=644 - **Screened:** n=416 - **Baseline:** n=330 - **Follow up:** n=194 intervention, n=97 control, 30% unable to contact |
| **Kolovou, V., et al. [13]** | UK | RCT | Awareness and Beliefs About Cancer (abacus3) | Cancer | Adults living in socioeconomically deprived areas | - **Goal:** n=107 venues, n=246 participants - **Screened:** n=448 - **Baseline:** n=39 venues, n=234 (n=117 intervention, n=117 control) - **Follow up 1:** 90.5% at 2 weeks - **Follow up 2:** 85% at 6 months |
| **Grazioli, V.S., et al. [14]** | Switzerland | Questionnaires | Harm-reduction drop-in center | Substance use | Socially marginalized alcohol and other drugs users | - **Goal:** n=132 - **Baseline:** n=85 - **Follow up 1:** n=72 at 1 month - **Follow up 2:** n=63 at 6 months |
| **D'Hooghe, S., et al. [15]** | Belgium | Walk-along interviews and focus group | CIVISANO-project | Physical activity | Persons in socioeconomically disadvantaged situations in peri-urban areas | - **Goal:** n=12 municipalities, n=127 individuals - **Baseline:** n=2 municipalities, n=38 individuals - **Follow up 1:** n=22individuals |
| **Milcarz, M., et al. [16]** | Poland | Cross-sectional study | PL-13 Program, “Reducing Social Inequalities in Health” | Reducing tobacco use | Adult population of social assistance beneficiaries/beneficiaries of government welfare assistance from Piotrkowska district | - **Goal:** n=3636 - **Baseline:** n=1817 - **Follow up 1:** n=1617 |
| **Sinclair, A. And H. Alexander [17]** | Scotland | Interviews | The Keep Well initiative | Coronary heart disease | People who had failed to attend a health check | - **Goal:** n=42 - **Baseline:** n=30 |
| **Craddock, E. [18]*** | UK | Semi-structured interviews | NA | Health and wellbeing | Members of the Women’s Health Network (WHN) in Bradford who represent marginalized women in the area | - **Baseline:** n=12 |
| **Mueller, J., et al. [19]*** | UK | Semi-structured interviews | “Let’s be clear, get it checked!’ | Cancer | Those involved in intervention’s management and delivery, revealing their perception of | - **Baseline:** n=10 |
| **Bodewes, A.J. and A.E. Kunst [20]*** | Netherlands | Primary: Analysis of recruitment strategies  Secondary: low budget surveys | Low budget health surveys |  | Moluccans | - **Approached:** n=19 Moluccan districts |
| **Ridley, J., S. Hunter, and A. Rosengard [21]*** | Scotland | Focus groups and in-depth interviews as part of larger study (national research program to evaluate major developments in mental health legislation in Scotland following the implementation of the Mental Health (Care & Treatment) (Scotland) Act) | The Mental Health (Care & Treatment) (Scotland) Act 2003 implemented in 2005 | Mental health | Mental health carers (experiencing mental health challenges), i.e. Supporting relatives who were under different compulsory measures and so had experience of new procedures under the Act | - **Baseline:** carers in n=3 contrasting Health Board areas and the State Hospital (hereafter referred to as Areas 1–4) at two stages approximately 12months apart. - **Baseline:** n=16 in focus groups, n=5 interviews - **Follow up 1:** n=15 carers in n=4 focus groups, n=5 individual interviews |
| **Bysted, S., et al. [22]*** | Denmark | Recruitment strategies | Face to face recruitment for intervention (health promotion and disease prevention) | Health promotion | Residents from disadvantaged neighborhoods, adults with mental, social or physical issues | - **Goal:** n=3 neighborhoods: N1 (n=101), N2 (n=143), N3 (n=60) - **Baseline:** N1 (n=35/101) doors visited, N2 (n=42/143), N3 (n=33/60) - **Follow up 1:** N1 (n=1), N2 (n=3), N3 (n=3) appointments for health consultations made - **Follow up 2:** N1 (n=8), N2 (n=8), N3 (n=1) realist interviews |
| **Lewis, S., et al. [23] *** | UK | Qualitative data (1^st^ phase) of the Communities in Control study | Big Local *(ongoing)* | Public health decision making | Residents from disadvantaged neighborhoods | - **Approached:** n=10 geographically diverse Big Local areas, - **Baseline:** n=440 hours observations, n=138 interviews (residents, workers and other stakeholders), n=18 focus groups, n= 30 BL plan documents |

**Table 2.** Descriptions of seldom heard groups, and categorized factors that contribute to their status as “seldom heard” groups, in article text

| **Main description of target group [reference]** | **Categorized reasons that group is considered seldom heard** |
| --- | --- |
| Socially disadvantaged women participating in BIG classes [1] | **Social status** and **Socio-demographic/economic:** characterized by, e.g., having a low household income, having a migration background, being unemployed, relying on welfare aid, and/or being a single mother (also **Current situational constraints/factors** and **Gender** and **Social services/resources** and **Immigration status**)  **Occupation:** unemployed  **Gender:** being single mother, traditional roles of women, culturally inappropriate facilities (such as mixed gender classes and male instructors) and the traditional role of women being mainly responsible for childcare and household duties  **Participants (or not) of existing intervention or program:** women participating in BIG classes (BIG project was initiated in 2005 by the Department of Sport Science and Sport, Friedrich-Alexander-Universität Erlangen-Nuremberg)  **Immigration status:** especially for women with migration backgrounds, barriers to physical activity include communication issues, cultural norms/traditional roles of women, culturally inappropriate facilities (such as mixed gender classes and male instructors) and the traditional role of women being mainly responsible for childcare and household duties ...Limited language skills as barrier, as they do not understand the language spoken at PA offers  **Current situational constraints/factors:** a lack of time  **Health and health behaviors:** physical and mental illness  **Negative beliefs or expectations:** a lack of self-confidence, or low motivation  **Socio-demographic/economics:** having low educational levels, financial problems |
| Two cohorts of marginalized men, consisting of two counties in Ireland, i.e. Men's Sheds members [2, 3] | **Gender and** **Difficult-to-reach/engage**: ‘hard-to-reach’ (HTR) groups of men (i.e. those who are unemployed, socially disadvantaged, isolated and have low educational attainment, marginalized (also **Social status/participation** and **Socio-demographic/economics),** these same groups are frequently the least likely to engage with health promoting initiatives that are traditionally regarded as an inaccessible of HTR cohort of men, ‘at risk’ (in terms of health status), men’s avoidance of health promotion and health services as a consequence of aligning to more traditional traits of masculinity such as stoicism, self-reliance and competitiveness (also **Health behaviors** and **Healthcare services)**  **Age:** more traditionally considered both hard-to-reach’ (‘HTR’), that is, those who are older, retired (also **Socio-demographic/economics)**  **Participants (or not) of existing intervention or program:** Shedders who participated in a structured health promotion program (Shed’s for Life)  **Social services/resources:** global gender equity policy still often fails to acknowledge men or else to position men and masculinities in a negative way, thereby creating challenges in translating knowledge into practice  **Healthcare services:** currently health services are not adequately versed in gendered approaches that effectively engage men |
| Socially disadvantaged young to middle aged men who are seldom in contact with health services [4, 5] | **Age** and **Gender**: young to middle-aged disadvantaged men  **Residence/housing location:** lived in areas of high social deprivation  **Disadvantaged:** socially disadvantaged  **Healthcare services:** majority never access professional help  **Social services/resources** and P**articipants (or not) of existing program):** the uptake of public health interventions among disadvantaged men is low. interventions were developed for and are usually delivered in healthcare settings. The group who binge drink most frequently, young to middle-aged disadvantaged men, are seldom in contact with health services. They are therefore much less likely to be reached by current initiatives to tackle excessive drinking  **Disadvantaged:** socially disadvantaged individuals, young to middle-aged, disadvantaged men, disadvantaged and low-income groups (also **Socio-demographic/economics)**  **Hard-to-reach/engage:** hard-to-reach population |
| Farmers and agricultural workers, individuals from the farming community, individuals who come into contact with people from the farming community in Scotland [6] | **Occupation and Current situational constraints :** farmers experiencing high stress levels due to financial challenges. Farming is a unique mix of intertwined circumstances that are potentially hazardous to mental health, such as social isolation, long hours, variable income due to circumstances out with one’s control such as the weather, and distance from services and may be less preventative in an occupational setting). Farmers often find it difficult to take holidays...suffer long-term exposure to pesticides and other chemicals, which may be linked to health conditions including problems with the central nervous system...New technology, regulation, administration, and digitalization bring challenges for mental health (Coping with paperwork ranked one of the highest stressors for farmers, who perceive a lack of support dealing with bureaucracy, ’Technostress’ = having to adapt to new information and communication technology (ICT)  **Gender:** farmers are more likely to enact more traditional views of masculinity such as stoicism and self-efficacy. Women in farming have different stressors (sometimes have to take on more work outside the farm to bring in additional income as farming incomes drop, and take on both the stress of the farm and additional stress of managing the wellbeing of family members)  **Negative beliefs or expectations:** concerned about stigma around mental health. Stigma play a negative role in accessing mental health help (depression being seen as a ‘weakness of character’), believing that professional help inadequate with long waiting times (also **Healthcare services)**  **Health behaviors** (outside of formal healthcare services): experiencing long-term back pain was a predictor of suicide...Increased alcohol use also increases the likelihood of mental distress  **Age:** Older male farmers less likely to access help for their mental health (also **Healthcare services**)  **Residence/housing location:** rural young men are less likely to seek mental health help from their GP compared to urban young men (also **Healthcare services**). Living in rural areas make it difficult to access help due to a variety of factors (lack of awareness of services available, cost of travel, lack of privacy, no local mental health services, long travel times, lack of choice of health providers, increase reliance on local GPs. Living in rural areas: increases the visibility and makes it more difficult for people to access help without the fear of stigma, compared to those in more urban areas (also **Healthcare services** and **Current situational constraints)**  **Healthcare services:** have lower rates of health seeking behavior for mental illness than the general population. Clinicians often lack training around farming as a sub-culture, for example that farmers cannot take extended periods of time off work or the difficulty of separating work and home life, Farmers are often reluctant to seek help so clinicians must be aware that conversations might occur indirectly whilst they are consulting a doctor for another reason. Seasonal farm workers often lead a transient lifestyle that makes it difficult to access continuity of care… independence as barrier to seeking help |
| Workers living or working in an area of North London [7] | **Ethnic background:** people of Turkish, Somali and White (British or Irish) backgrounds  **Other adjectives:** disadvantaged groups, isolated population  **Occupation:** workers  **Socio-demographic/economics:** primarily from lower socio-economic neighborhoods (**Residence/housing location)**  **Residence/housing location:** lower socio-economic neighborhoods, living or working in an area of North London |
| Women migrants in a Swedish context from the co-creative lab [8, 9] | **Immigration status, Ethnic background** and **Personal history**: migrants from Middle Eastern countries, the majority speaking Arabic, but also Persian occurred in the population (though the group was open to women with any ethnic origin). During transit and post-migration (adverse experiences and situations, inequitable distribution of power, income (also **Socio-demographic/economics)**, and other determinants of health, social isolation, or exclusion from the host society...migrants are exposed to inequitable distribution of resources, social identities of the study population as those that usually are deprived of privileges due to their belonging to an ethnic minority. Also, many of them had migrated from countries afflicted by armed conflicts, meaning that the population may be fulfilling another social identity, disability, due to mental issues because of traumas (35). Disability in the shape of stress and long-term pain had previously been acknowledged in the women’s health group. The comorbidity of physical and mental issues has previously been described among migrants coming from countries afflicted by armed conflicts, Previous research has also shown that low social support (also **Social status/participation)** among migrants was associated with a higher degree of pain (also **Health and health behaviors);** Research has shown that around 20% of newly arrived migrants from Syria felt excluded and isolated (also **Social status/participation)** in the Swedish society, and this was associated with mental issues like PTSD, anxiety, depression, and low subjective well-being (also **Health and health behaviors**)  **Residence/housing location:** socially deprived area where parts of the population may be marginalized (classified as a socially vulnerable area, According to the Swedish police authority, a socially vulnerable area is defined as an area with a population with low socioeconomic status and widespread criminality that affects the local society) (also **Socio-demographic/economics** and **Current situational constraints)**  **Current situational constraints/factors:** Many stressors, such as housing or work  **Participants (or not) of existing intervention or program:** migrant women study population from the co-creative lab focusing on women’s health  **Gender:** women, social identities of the study population as those that usually are deprived of privileges due to their gender (also **Social status/participation** and **Current situational constraints/factors:)**  **Socio-demographic/economics:** Poor socioeconomic status, social identities of the study population as those that usually are deprived of privileges due to having low socioeconomic status (also **Social status/participation** and **Current situational constraints)**  **Social status/participation:** previous research has shown that around 55% of Syrian newly arrived women migrants had a weak social support in Sweden, Migrant women have previously scored lower in measures of self-esteem compared to non-migrants, which has been suggested to have an association with the triple discrimination they might experience  **Healthcare services:** health care has had difficulties achieving healthy lifestyle changes within the population ...women migrants have difficulties engaging in PA...lower mental quality of life, as well as lower self-esteem. The power balance between healthcare personnel and patients in general has previously been described as patients having a threefold subordination: the institutional, the existential, and the cognitive - they are lowest in the institution hierarchy, vulnerable due to disease, and least knowledgeable compared to the personnel they meet in the health care. there has been a focus on person-centered care in Sweden, aiming to include the perspective of the patients more – but patients belonging to the general population in Sweden are not satisfied with the care they get. Target population may feel discriminated against due to being women and belonging to a minority ethnic group  **Personal history** and **Current situational constraints/factors:** lower self-esteem associated with experiences of discrimination on three grounds (gender, social status, and ethnicity - various social identities occur simultaneously lead to identities experiencing oppression)...A low belief in the future widespread among the population in such areas (related to risk factors such as unemployment, and worse living conditions, or to global conflicts being present in the local environment, increasing disturbances)...may not be represented in the political system and public debates, a low degree of trust between residents may impair the collective capacity (also **Negative beliefs or expectations** and **Social status).** |
| Farmers who took part in a personalized telephone coaching as part of the ‘With us in balance’ project [10] | **Occupation:** farmers, long working hours, uncontrollable weather conditions while often living in remote areas  **Participants (or not) of existing intervention or program:** farmers who took part in a personalized telephone coaching as part of the ‘With us in balance’ project.  **Residence/housing location:** live in underserved areas, uncontrollable weather conditions while often living in remote areas  **Healthcare services:** low help-seeking behavior  **Negative beliefs or expectations (technology concerns):** data security worries, lack of personalization, insufficient computer skills, or internet availability |
| Women from an area of disadvantage [11] | **Gender:** women  **Residence/housing location:** from an area of disadvantage, residence of a "regeneration area" (a place of violence, social dysfunction and danger: a troubled region of Ireland (Limerick) (also **Current situational constraints/factors)**  **Current situational constraints/factors:** stigmatization, Limerick residents experienced prejudice, stereotypes, and inequity in access to social and financial services...Having endured such monikers as ‘Stab City’ and ‘troubled’, resistance to being saddled with yet another reminder of the town’s socio-economic status |
| Residents of economically deprived boroughs in London [12] | **Socio-demographic/economics:** economically deprived  **Social status/participation:** disadvantaged  **Ethnic background:** ethnic minority groups  **Residence/housing location:** patients from disadvantaged communities, economically deprived boroughs in London  **Representation in research:** rarely represented in research |
| Adults living in socioeconomically deprived areas [13] | **Hard-to-reach/engage:** a historically “hard-to-reach” group. The recruitment and retention challenging in health research (participant samples rarely reflect realistic socioeconomic deprivation patterns) (also **Representation in research,** and **Socio-demographic/economics** and **Social status/participation)**. Traditional recruitment strategies/primary and secondary care settings miss the populations who do not regularly engage with these services (also **Healthcare services)**  **Residence/housing location**: adults living in socioeconomically deprived areas, communities (prolonged time to symptom presentation due to barriers of lower cancer symptom awareness and negative beliefs about cancer (also **Health and health behaviors** and **Negative beliefs or expectations**)  **Negative beliefs or expectations:** Evidence suggests that low awareness of cancer symptoms, fear of cancer or fatalistic beliefs about cancer and concerns over wasting the doctor’s time are higher in lower socioeconomic groups result in delayed help seeking in turn leading to late stage diagnosis and lower uptake of cancer screening (also **Healthcare services)**  **Healthcare services:** Barriers that prevent participation in these settings (deteriorating health, lack of transport, difficulty accessing services and poor understanding of written recruitment materials) (also **Current situational constraints/factors**) |
| Socially marginalized alcohol and other drugs users [14] | **Marginalized:** Socially marginalized individuals, characterized by the European Network of Social Inclusion and Health as “individuals, groups or populations outside of mainstream society, living at the margins of those in center of power, of cultural dominance and economical social welfare” (also **Socio-demographic/economics**)...Social marginalization (an umbrella term to refer to individuals who regularly use publicly funded clinical and social services (i.e., shelters, drop-in centers, case management, and medical and psychiatric centers). most socially marginalized individuals do not present for widely available abstinence-based treatments (only 7% with substance use disorders in Zurich, Switzerland reported having attended abstinence-based treatment over the past 6 months) (also **Healthcare services)**, marginally housed or homeless individuals (also **Residence/housing location)**, social assistance recipients, and drug users (also **Health and health behaviors)** |
| Persons in socioeconomically disadvantaged situations in peri-urban areas [15] | **Disadvantaged:** socioeconomically disadvantaged situations  **Residence/housing location** and **Current situational constraints/factors:** peri-urban areas, often more dependent on their direct environment due to their smaller activity radius. socioeconomic disadvantage phenomenon less visible in peri-urban and rural areas (challenges regarding accessibility, physical and social mobility, e.g. limited transport opportunities and inaccessibility of infrastructures, lacking city economics of scale or budgets to address these issues) (also **Healthcare services** and **Social services/resources)**  **Participants (or not) of existing program:** less likely to engage in recreational walking  **Negative beliefs or expectations:** tend to perceive environment negatively (perceived environmental factors better predictors of self-reported health behaviors, such recreational walking |
| Adult population of social assistance beneficiaries/beneficiaries of government welfare assistance from Piotrkowska district [16] | **Social status/participation:** socially-disadvantaged people, the definition of socioeconomically disadvantaged, population covers persons with the minimum income not greater than 634 PLN per month for single persons and 514 PLN per month for family members. poverty threshold as adopted by the social assistance institutions was used (incomes lower than specific poverty lines as identified in the Social Assistance Act), as reflected in the applicable regulations (also **Socio-demographic/economics)**  **Residence/housing location:** resided in the Piotrkowska district. Piotrkowska district as 11th among all 314 rural districts with the lowest indicators of social development in Poland  **Social services/resources:** received aid offered by the local social assistance organizations. The right to benefits under the system is given to individuals and families who are unable to cope with difficult life situations using their own empowerment, resources and abilities. registered with the local government welfare assistance institutions and entitled to receive social aid. they often have limited access to healthcare (also **Healthcare services)**, defined here as a place other than traditional health services that provides antismoking activities and interventions  **Current situational constraints/factors:** low income, poor housing and unemployment (also **Socio-demographic/economics)**, financial pressure and stress, parental and peer example, nicotine exposure during childhood, targeted and more intensive marketing by the tobacco industry, as well as a lower likelihood of working indoors (also **Occupation),** target group for tobacco industry targeting this price-sensitive population through promotions and price discounting, as well as undermining policy efforts (also **Social status/participation)**  **Socio-demographic/economics:** Unemployment, inability or unwillingness to undertake a job is the key poverty factor (also **Occupation),** barriers to disadvantaged group participation in medical research including difficulties in identifying and sampling the hidden populations (also **Representation in research)**, mistrust of medical research and the researcher (also **Negative beliefs or expectations)**, cultural or language barriers (also **Ethnic background)**, low literacy, and low education **(**also **Participation (or not) in existing program):** Some cultural and educational barriers among disadvantaged populations can create a situation in which there are different attitudes towards tobacco smoking and its health consequence barriers in their sampling, recruitment, participation, and retention, and samples based on the general population were rarely sufficient to adequately examine outcomes according to social group (also **Health and health behaviors** and **Social status/participation**) |
| People who had previously failed to attend a health check [17] | **Socio-demographic/economics:** economically deprived  **Participants (or not) of existing intervention or program:** non-attendance at screening or general practice appointments due to forgetfulness, fear of outcomes, ambivalence towards purpose, unwillingness to commit time or finances to such activities, and the perceived low importance of such appointments/low priority of a health check (also **Negative beliefs or expectations** and **Healthcare services**)  **Age** and **Gender:** Non-attenders have also typically been shown to be young, male and from a lower socio-economic background (also **Socio-demographic/economics)**  **Hard to reach/engage:** ‘hard-to-reach’ is often used within the service and marketing sectors with reference to individuals who prove difficult to involve in or who do not access the services that are available to them (also **Healthcare services);** more specifically: lack of social confidence (also **Social status/participation)**, distrust of authorities, fear of the unknown and communication difficulties (also **Negative emotions or expectations)** |
| **Article described Interviews:** members of the Women’s Health Network in Bradford  **Target group:** marginalized women in the area [18]* | **Representation in research:** Research in healthcare has historically neglected to take into account women’s lived experiences of health and illness (Yentl syndrome), underrepresented in medical trials  (also **Healthcare services):** Misdiagnosed and poorly treated unless their symptoms mirror those of a man, traditional patriarchal roots of medicine (also **Health and health behaviors** and **Social status/participation)**  **Social status/participation:** Refers to women who face axes of multiple oppression alongside sex such as ethnicity, socio status, sexuality, disability, age migrant status (also **Gender**, and **Age** and **Ethnic background)** |
| **Article described Interviews:** volunteers delivering the intervention or participating in the steering group.  **Target group:** vulnerable groups from areas of high socio-economic deprivation [19]* | **Healthcare services:** people delay presenting to health services with cancer symptoms due to lack of awareness (also **Health and health behaviors)**, particularly in areas of high socio-economic deprivation (also **Socio-demographic/economics)**  **Socio-demographic/economics:** Deprivation has also been linked to increased exposure to predisposing factors, including poor lifestyle choices, unemployment, low education levels and low housing quality (also **Health and health behaviors** and **Residence/housing location** and **Current situational constraints/factors)**  **Ethnic backgrounds:** ethnic minority groups, referred to as ‘vulnerable groups’.  **Current situational constraints/factors:** people with learning disabilities, referred to as ‘vulnerable groups’. |
| **Article described analysis:** recruitment strategies  **Target group:** Moluccans, aged between 30 and 65 years, living in Moluccan districts in Netherlands [20]* | **Representation in research:** most of the research on health of minorities focuses on the largest minority groups of Moroccans, Turkish and Surinamese. One hard-to-reach minority group has been largely ignored until now: the Moluccans (also **Ethnic background)** wait-and-see attitude restricts their close involvement in research (also **Health and health behaviors**)  **Personal history:** History of being exiled after serving the Dutch army and being transferred to the Netherlands: political history has left its scars within the community, resulting in distrust towards the Dutch government and also towards non-governmental organizations  **Socio-economic or demographics:** lower educated and have lower-ranking occupations than the Dutch autochthonous population  **Residence/housing location:** “Moluccan districts” located in the outskirts of central Netherlands  **Culture:** culture is one with a strong adherence to traditional values, social hierarchy, family bonds and Malay language. Privacy is paramount and health is not discussed outside of the immediate family |
| **Article described analysis:** Mental health carers (carers of those with mental health conditions)  **Target group:** Mental health carers supporting relatives who were under different compulsory measures and so had experience of new procedures under the MHCT Act [21]* | **Occupation:** informal occupation as an informal carer  **Health and health behaviors:** Mental health problems challenge such models of care; individuals with mental health problems have fluctuating needs that differ from those associated with physical frailty and dependence, thereby creating dissonance for those involved. Caring is described as a ‘burden’ and in the mental health field, this refers to the stress family members experience when supporting someone with severe mental health problems (also **Current situational constraints/factors**). the longer the caring role persists, the less optimistic carers tend to be about the support offered and many experience an adverse impact on their mental and physical health (also **Negative beliefs or expectations),** e.g. “Coping, often with little or no support, with the traumatic and stressful impact of compulsory detention of their relative, had taken significant toll on the carers’ physical and mental health.” A female carer reported “I was a nervous wreck. My GP had phoned the psychiatrist because she thought I was going to take my life”. carers interviewed were depression and suicidal thoughts as they struggled to prevent health crises in households with multiple and complex needs (also **Healthcare services** and **Health and health behaviors)**  **Social status/participation** and **Psychosocial:** Mental health carers, especially those caring for relatives subject to compulsory care and treatment, often feel overlooked and marginalized, caring in complex circumstances with little or no professional support. Their occupation and rights, for example as a Named Person, are not validated (i.e. known) by some healthcare providers making it more difficult for them to be “seen and heard”, undervalued. Felt powerless and were often supporting their relatives without adequate support. (based on results) Familial relationships can be damaged by the bureaucratic responsibilities, being a Named Person (i.e. carer with access to all health information), based on disagreements with their relatives. It was described as “a difficult line to tread”. Their level of control and power could be taken away, e.g. Named Person, if their relative becomes dissatisfied (also **Negative beliefs or expectations)**  **Social services/resources:** lack of access to appropriately targeted information and advice; lack of recognition for carers; and a lack of access to responsive services. “Many in the sample did not believe they were entitled to any services for themselves because of the varying intensity and kinds of support required when caring for someone with fluctuating mental health problems. What they were agreed upon was that trying to get help and advice for themselves was ‘an absolute nightmare’, or at the very least, ‘an uphill struggle’. A general lack of support, compared to that received by carers of other people with disabilities, has been reported elsewhere, for example, by Australian carers”  **Occupation** and **Socio-demographic/economics:** Employment/financial status  **Hard to reach:** caring for people with mental health problems can be a ‘hidden’ role bound by complex and sensitive relationships, it was recognized that such carers could be ‘hard to reach’.  **Residence/housing location:** practical barriers such as geographical dispersal impacted significantly for those carers residing across Scotland who were trying to maintain involvement with individuals held at the State Hospital (also **Current situational constraints/factors)** |
| **Article described analysis:** recruitment strategies  **Target group:** Residents living in disadvantaged neighborhoods, i.e.  Adult residents with mental, social or physical health issues and/or residents lacking social or economic resources [22]* | **Healthcare services:** participate less in health services, participate less in prevention program, they are impeded by lack of capacities and resources, including time and money required to access activities (i.e. prevention programs)  **Residence/housing location** and **Socio-demographic/economic**: geographically delimited areas with a large share of people with low socioeconomic status (SES), for example, unemployed, low educational attainment and low income  **Social services/resources:** For these specific neighborhoods, 40%–60% are receiving public benefit compared to 27% in the municipality in general and they house many different ethnicities, people living alone or being a single parent (also **Current situational context** and **Ethnic background**)  **Socio-demographic /economic:** People living in public housing areas are more often unemployed, have lower educational attainment and lower income |
| **Article described analysis:** 1^st^ phase of the Communities in Control study interviews (residents, workers and other stakeholders), focus groups, plan documents  **Target group:** residents from disadvantaged neighborhoods [23] * | **Residence/housing location:** residents of disadvantaged neighborhoods  **Social status/participation and Social services/resources:** living in disadvantaged circumstances  **Current situational constraints/factors:** inequalities in collective control amongst social groups may be a driver of health inequalities (also **Health and health behaviors)**  **Social status/participation:** this field is characterized by conceptual confusion (Overlapping and sometimes contradictory terms have found their way into the policy lexicon including, in particular: community-centered, place-based approaches, asset-based community development, connected communities, community resilience, community empowerment, and collective control.), which risks undermining the impact of these initiatives, Participation alone is insufficient if strategies do not also build capacity of community organizations and individuals in decision-making and advocacy, the reason for the apparently poor outcomes at the community level of previous English ABI New Deal for Communities was that there simply were not enough involved individuals. (also **Representation in research)** local infrastructural barriers that preceded the introduction of BL, or challenges arising from a community’s cultural, social or economic diversities residents identified socially or culturally with sub-areas rather than the whole BL area (also **Socio-demographics/economic)**, or boundary changes predating BL have disrupted local place-based identities (also **Residence/housing location)**, e.g. residents did not identify with a BL area because of assumptions of socio-economic difference or “territorial stigma” (also **Negative beliefs or expectations),**  **Negative beliefs or expectations:** previous experiences of failed local regeneration initiatives  **Participant (or not) in existing program:** venues were unsuitable to some residents, infrastructure (e.g. road system cutting through the intervention area so that some residents choose to volunteer in a sub-community as opposed to the whole intervention community) (also **Residence/housing location)**, Scheduling of partnership meetings can make participation seem impossible, Barriers to empowerment and collective control over decisions and actions - including external or political interference - that have afflicted previous place based initiatives (also **Current situational constraints/factors)**  **Current situational constraints/factors:** struggling to pay rent or afford food (also **Socio-demographic/economic**), parental responsibilities/priorities etc. |

**Table 3.** Recruitment team and material

| **Reference** | **Recruitment personnel**   - [Role/activities] | **Study information/invitation and recruitment materials** |
| --- | --- | --- |
| **[1]** | **Local project coordinators, trainers, and other BIG stakeholders (e.g., longtime participants and cultural mediators who support the BIG project at each site):**   - Informed potential participants about the details of the interview study by phone, e-mail, or personal meetings - Subsequently asked to present them to the class participants   **Research team:**   - Advertised in BIG classes - Collected information from those interested and contacted them later - Informed participants about purpose of study and consent details | - Phone - E-mail - Personal meetings - In-person advertising |
| **[2]** | **Research team and members of The Irish Men’s Sheds Association (IMSA):**   - Conducted recruitment carried out through an expression of interest process targeting each Shed and a series of Shed visits | - NA |
| **[3]** | **[No specific person mentioned]:**   - Sending all sheds in targeted counties in Ireland a promotional package and inviting them to register an ‘expression of interest’ in participating in shed’s for life (SFL)   **First researcher and members of the IMSA:**   - Visited sheds that expressed interest in participating to discuss the SFL process and to recruit individual shedders to both the program and the evaluation - Objective to deliver SFL in a diverse range of shed settings (small/large, urban/rural) - To build a sense of rapport and trust, and to assess the shed environment’s suitability to participate in the program (including adaptations needed to facilitate this)   **Member of the research team:**   - Explained details of the research through verbal and written instruction, collected informed written consent   **Irish heart foundation nurse:**   - Was a longstanding partner of the IMSA whose staff are vastly experienced in engaging populations of marginalized men - Ran screening elements of SFL - Delivered health check directly in the shed setting during routine shed hours   **SFL partners:**   - Adequately insured and qualified to run elements of SFL and - Engage in a screening process with participants to assess their ability to partake in the intervention for safety purposes | - Promotional package: inviting them to register an ‘expression of interest’ - Written and verbal details of the research: description as a program ‘for shedders by shedders’ - In-person presentation |
| **[6]** | **“The group”:**   - Advised on how best to reach farmers with fliers / leaflet etc. - Advised about images used in written materials | - Electronic and hard copy recruitment materials and ‘Participant Information Sheets’ emailed or posted ahead of the interview [copies provided in publication] |
| **[7]** | **[No specific description of person]**   - In-person direct recruitment in ‘natural smoking settings’ including bars and cafes   **[No specific description of person]**   - Ten replacement smokers recruited for post-legislation interviews, matched as closely as possible with respect to age, sex and ethnicity to those lost to follow-up   **Community contacts**   - Aided in recruitment | - NA |
| **[4]** | **3 general practices:**   - Provided letters of invitation   **Research fellow:**   - Telephone discussion with potential participants   **The many community groups in Dundee:**  Helped to recruit the participants | - Participant information leaflets by post |
| **[8]** | **Representatives from academia/Malmö University, voluntary organizations (The Red Cross, Save the Children, Skåne Sport Federation), the business sector (pharmacy, housing company, technology company, oral health company), and the public sector (Region of Scania, Malmö City, Finsam – a state financial coordination covenant in rehabilitation)**   - Strategic steering group   **Lay health promotors/Citizens of the community:**   - Advisory role in the steering group - As health promoters chosen by the citizens to represent the community in the CBPR planning and could be seen as facilitators for participant recruitment - Kept up the contact between participants and researchers - Reached out to and recruited new participants and informed about activities - Distributed written invitations to women in the co-creative lab   **Researchers:**   - Visited the women´s area and the place where the study was conducted on several occasions before starting the research to build trust - Invited Women from the community health program to take part of the research and could communicate in their own language - Provided oral information about research study and answer any questions | - Written invitations to meet the researchers to receive oral information about the research study (and in own language) |
| **[9]** | **Citizen health promoters/ The Lay health promoters (LHP):**   - Volunteered during workshops to help co-ordinate the activities within the program - Later employed part-time by program - Facilitated participant recruitment, and language interpretation - Build trust between the researchers/stakeholders and the citizens - In contact with those interested to participate prior to the day of data collection | - In-person presentation about data collection - Invitation given during preceding health promotion activities (translated by the LHP into the women’s language) |
| **[10]** | **German Social Insurance for Agriculture, Forestry and Horticulture (SVLFG2) (TEC-A):**   - Sent personal invitation letter, or via consultation - Provided general information, e.g., on their website and via staff members | - Information via personal invitation letter - Information via consultation - General information (on their website and via staff members) |
| **[11]** | **School staff:**   - Provided information sheet and gathered informed consent through their role as choir administrators   **Gatekeeper (may also be school staff):**   - Liaise with the choir | - Information sheet offered to choir participants |
| **[12]** | From **protocol**:  **GP practices:**   - Referral will be via various methods including: (1) a simple fax referral form similar to those that were used in the pilot work (study center staff will then proactively contact potential participants); - (2) telephone referral - (3) providing potential participants with the study contact details via ‘tear off’ pads, mailshots to patients with BMI of 30 or more (see details below), and in-practice promotional materials)   **(4) patients contacting the study center:**   - Via ‘word-of-mouth’   **(5) local advertising:**   - If necessary, to boost recruitment   **Routine practice staff:**   - Search of computerized GP records to identify potential participants (e.g. People with a inclusion BMI) - Generation of GP letters informing participants of the option of participating in this project)   **Trained person delegated by the investigator:**   - Obtained written informed consent as documented in the site delegation log, prior to any participation/study specific procedures)   **Reported**  **Two large local GP practices (one in each borough)**   - Recruitment via fax referrals - Posters - Flyers - Mailshots)   **Four neighboring GP practices**   - Via referral   **Local community venues:**   - Posters - Leaflets   **Local press**   - Three advertisements | **From protocol**   - Pre-screening for eligibility by phone - Participant information sheet/ study details - Screening visit:   **Reported**   - Information about the study (via telephone at first contact, and were screened for eligibility) - Participant information sheet, baseline questionnaire, and an invitation to the first study session a few days later (eligible participants were sent (via post or email). |
| **[13]** | **Lay advisors (two based in South Yorkshire and one based in South East Wales):**   - Undertook recruitment - Responsible for communication with key stakeholders - Identification of eligible venues - Contacted Individual healthcare and community venues via emails and phone calls to introduce and invite participation in the trial as a recruitment venue. - Approached local councils governing the most deprived communities intending to start a snowball effect in community settings - Liaising with local gatekeepers/stakeholders in healthcare setting - Where willingness to take part was indicated, the lay advisors would visit and meet local gatekeepers at the venues to check venue eligibility– chiefly, a private room to conduct the intervention and a reliable internet connection needed for the online health check. - Organizing recruitment days - Use a checklist to assess eligibility - Enrolling and randomizing participants - Kept a database of all requests to community and healthcare venues, detailing contact attempts made by the trial team and the postcode of the venue, i.e. Reflections of each visit to a recruitment venue using a standardized proforma. These records included details of the day of the week, time of arrival and departure, the type of recruitment session arranged (opportunistic, pre-booked appointments or both), the total number of people recruited at the venue and an estimate of the average footfall at the venue while the advisor was present - Searched and opened new healthcare and community settings regularly throughout the recruitment window in order to secure further recruitment days and avoid recruitment stagnation. - Made Pre-booked appointments with interested individuals, led opportunistic recruitment by approaching individuals in a community or healthcare setting on set recruitment days agreed with the venue manager or other staff, assessed for eligibility   **Local stakeholders supporting the trial, i.e. Pharmaceutical Committees (Yorkshire), Clinical Commissioning Groups (Yorkshire) and Welsh University Health Boards (South Wales):**   - Sent an Expression of Interest form (to healthcare settings))   **Venue managers and key staff:**   - Visits before recruitment were used to establish rapport with venue managers and other key staff and encourage a collaborative approach to recruiting local participants. - Advised on the days when there would be maximum footfall, described the demographic profile of their visitors (e.g. Age and language). - Those who had capacity to support recruitment further booked provisional appointments with potential participants ahead of the advisors visiting for a recruitment day, i.e. Pre-booked appointments with interested individuals - Opportunistic recruitment will be used where local staff are unable to support the appointment system. In such instances, lay advisors will either visit venues a few days in advance to approach individuals and book them in for the upcoming recruitment day or approach potential recruits on the day or a combination of both, initially check participant eligibility verbally when individuals show an interest in the study and if confirmed as eligible will then proceed to full recruitment   **2 patient/public research partners in the trial team:**   - Provide support and knowledge at every stage of the research process. - One partner lives in one of the target recruitment areas, the other has significant family associations with the other area. Both have been affected by cancer - Were critical for the early development and set-up of the trial and have provided detailed input to the protocol development - Contributed to all public facing materials (i.e. Information booklet content and design, questionnaire design etc.), with national literacy levels in mind - Contributed to generate ideas on how best to engage the target population, support data interpretation as well as provide ideas on dissemination opportunities | - Patient-facing study materials, i.e. Participant information sheets, consent forms, and questionnaires - Abacus3 participant information booklet (provided after screening) - The English Index of Multiple Deprivation (IMD) or Welsh Index of Multiple Deprivation (WIMD): to identify participants by postcode |
| **[14]** | **Drop-in center and research staff:**   - Notified drop-in center attendees (as well as attendees at the sister site in the same complex) of the opportunity to participate in the program evaluation)   **Research staff (i.e., a psychologist):**   - Conducted information sessions with interested clients, during which she explained the purpose, content and structure of the program evaluation, participants’ rights, potential study-related risks, and informed consent materials | - Eligibility and screening questions - Informational flyers: posted at the drop-in center - Information sessions with interested clients |
| **[5]** | **Scottish primary care research network:**   - Identify potential participants , based on age and postcode   **General practitioners (GP) :**   - Invited them to take part in the study, i.e. Provided information letter - Identified from 20 GP practice lists/primary care registers (5 in each of the 4 centers   **Venues which the target group are known to frequent, such as charities that support the long-term unemployed, supermarkets, housing associations, and main shopping streets in disadvantaged areas:**   - Sampling at selected times of day and days of the week - This required initial fieldwork to identify appropriate venues and suitable times for recruitment - Venues will be visited at different times to prevent friendship groups from being included in the study)   **Research assistant:**   - Approach participants with invitation, i.e. Approach will be informal, friendly and sensitive to the men’s willingness to discuss participation)   Researchers:   - Contacted individuals who did not opt out of the study by telephone approximately 2 weeks after the GP letter was sent | - Information leaflet |
| **[15]** | (Protocol)  **Volunteer:**   - Depending on the covid-19 measures in place at the time of recruitment, a volunteer will either ring the doorbell and administer the questionnaire with a tablet or leave the paper questionnaire - Or a flyer (with web-link and QR-code to fill in the questionnaire online) in the mailbox, with a proposition to come back and pick it up a week later, - Or ask to drop the questionnaire in a predefined central mailbox in the municipality. - Participants will be asked at the end of each session if they know other persons that might be interested to join (Snowball technique).   **Participants themselves:**   - Additionally, after a respondent has completed the questionnaire, a snowballing technique (meaning that participants refer potential participants from their social circle to researchers) will be applied. - Participants will be asked at the end of each session if they know other persons that might be interested to join (Snowball technique).   **Members of research team:**   - Will be present in public places, at local social organizations and at local events and food distributions.   **Local organizations that support socioeconomically disadvantaged adults (local service centers or local health care centers):**   - Will invite them to participate.   **Municipality websites and local journal:**   - Announcements in the local journal and on the website of the municipality to increase visibility.   **[no specific person mentioned]**   - Furthermore, before participation, eligibility will be checked. - A few weeks later, participants were invited to engage in a focus group | - Municipality website - Local journal - Social media - Pre-distributed CIVISANO-questionnaire Door-to-door visits at pre-identified/purposefully selected local venues |
| **[16]** | **Local government**   - A list of the recipients was obtained - All those registered in the local government welfare assistance institutions who met the inclusion criteria and agreed in writing to participate in the study | - NA |
| **[17]** | **Nursing staff:**   - Sought verbal agreement for formal consent to be discussed   **Researcher:**   - Explained the purpose of the study and obtained formal signed consent for participation in the research | - Verbal agreement to be discussed for inclusion during the health check - Verbal explanation of study by the researcher |

*Extracted verbatim from the articles and summarized/paraphrased*

**Table 4.** Factors of retention and attrition

| **References** | **Facilitators of retention/engagement** | **Reasons for attrition or non-engagement** |
| --- | --- | --- |
| **[1]** | - Choice of engagement method - Cultural respect - Trusting atmosphere | - Time constraints as barrier to member checking - Experiences of women with short-term participation in existing program not considered |
| **[2]** | - Accounting for potential literacy issues - Gender specific approach and sense of safety - respecting the autonomy of Shedders to opt in/out of the program | - Capacity constraints of data collectors negatively affected follow-up rates - More effort than expected was needed for recruitment due to COVID - Respecting autonomy - Reduced resources for data collection due to COVID - Informality/sporadic attendance at venues negatively affected follow-up |
| **[3]** | - Gendered approach to design and delivery of program - Registration of potential participants as an ‘expression of interest’ emphasized participant control, autonomy and ownership - Building trust with target group prior to recruitment - Recruitment setting was familiar to potential participants - Description of program emphasized participant control, autonomy and ownership - Consideration of capacity and resource constraints of intervention settings (Sheds) - Measure of potential sample based on existing participation in existing program - Free health check as engagement strategy - tailored method of data collection as engagement factor | - Drop out measures not accurate due to informal nature of the Sheds - Non-representative sample - Underrepresented populations may feel stigmatized or labelled in this setting |
| **[4]** | - Message content - Follow-up messages were managed by system that iteratively checked if messages were delivered - Credible source of messages as engagement factor - Text intervention method as engagement factor - Unobtrusive data collection method - Representative sample | - Some messages to participants could not be tracked (limited measure of engagement and retention) - Some messages to participants were not delivered - Some participants did not respond to messages |
| **[6]** | - Advisory group in study planning and recruitment - Unstructured interviews as engagement factor - Brief interviews - Placing information where target group is | - Remote, as opposed to face-to-face, data collection due to COVID - Low number interviews with actual farmers as consequence of COIVID |
| **[7]** | - Interview schedule based on evidence and expert input - Replacements found | - Several lost to follow up due to mobility and “hard to reach” nature of population |
| **[8]** | - The CBPR approach - Location of the existing program - Involvement of other actors in planning/co-designed planning and specifically involvement of Community health workers - Migrant-woman founded program setting for recruitment and interviews - Personalized contact for follow up - Familiarity of study setting - Building trust prior to study - Communication in own language - Accounting for participants’ needs (i.e. time constraints)   *Background article: significant impact of study administration on engagement and retention/tailoring of the study experience to seldom heard group [24]* | - Lack of representative sample - Data collection time limited by participants’ time constraints |
| **[9]** | - Involvement of other actors in planning/co-designed planning and recruitment and building trust - Lay health promotors as liaison between researchers and participants during recruitment, ensuring trust - Participant representatives part of project planning - Data collection in areas participants were familiar/comfortable with - Reducing power inequalities between researchers and participants | - Power relations during group work involved “friction” (attempt to address issue: In the overall program all community members were involved in focus group session where they discuss collaboration and partnership related to culture and power every 6 month) - Recruitment based on LHP’s personal and capacity network may have led to missing potential participants - Loyalty to LHP may have skewed reality (more positive) of participant responses - Lack of certified translator as potential language barrier - Delay in data checking with participants - Same study population could not be reached for data checking due to interruptions (including COVID) |
| **[10]** | - Involvement of other actors in planning/co-designed planning - Participants prior experience with similar intervention to the study intervention - Insurance supported intervention as engagement factor - Personalization of intervention as engagement factor - Personalized support in addition to intervention offered - Continuity of intervention components - No cost to participants - Timing of intervention as engagement factor - Compensation | - Limited experience of study team members contributed to non-representative interpretation of interviews - No incentives were offered - Differing enrollment processes for certain participants - Limited sample size due to convenient and small sampling - Coaching was not presented at the same time for all based on participants’ time constraints - Did not reach meaning saturation due to sampling method and high satisfaction (but might not be reasonable for this study) |
| **[11]** | - Open inclusion criteria - Gatekeeper as liaison between researchers and participants | - Drop outs - “White coat” affect could have impacted responses - Limitation of literacy when participants answered questionnaire |
| **[12]** | - Compensation - Non-blinded intervention as engagement factor - Inclusiveness of trial as engagement factor - Camaraderie as engagement factor | - Lost to follow up - Methods for adjust for missing data were limited |
| **[13]** | - Lay advisors as recruiters - Community setting waived fees for use of facilities - Balance of recruitment venues - Clinical Commissioning Group provided payment to healthcare providers for their participation - Established rapport with recruitment venues prior to recruitment - Community members were patient/public research partners and contributed to public facing recruitment materials - Compensation | - Lost to follow up and lack of time and interest as reasons for non-participation - Those with highest need may not be recruited due to inclusion criteria |
| **[14]** | - Measured ability to consent prior to consent - Measures and timeline chosen with participants in mind | - Lost to follow up - Factors of attrition had to be factored into statistical analysis |
| **[5]** | - Final intervention message based on engagement and request they keep in touch - Follow-up assessment reminders sent - Compensation | - Incompatibility between strategies for minimizing impact of data collection methods on outcomes and increasing retention |
| **[15]** | - Setting selection based on census-like statistics and feasibility of researcher travel to gather data - Age inclusion criteria based on population - Being cognizant about potential stigmatization by not using certain terms to describe population - Language used for recruitment carefully vetted - Recruitment venues and strategy in areas where target group frequents - Compensation | - Illness, lack of interest, fear of COVID and lack of time as reasons for attrition - Self-selection bias amongst participants - Recruitment limited due to COVID - No incentives for quantitative part - Inability to account for all challenges of target group |
| **[16]** | - Face-to-face interviews reduced non-response rate - Use of local government records to identify potential participants | - NA |
| **[17]** | - Participants’ preferences for participation method - Short interviews based on typical time constraints of potential participants - Case sampling was appropriate for target population - Reassurance of representative sample | - Potential participants were previously non-attenders which limited expectations of reliability - Declined to participate due to lack of time to participate in interview |
| **[20]*** | - Compensation - Direct recruitment, door to door and announcement letter - Involving key informants in the research process | - Low response rate attributed to culture of individualism, privacy, value of personal contact over non-personal contact methods, and distrust - Limited time, funding and experience of researchers |
| **[21]*** | - NA | - Unsuccessful recruitment strategies by partners |
| **[23] *** | - Tying community events with recruitment - Flexibility and realistic expectations of involvement | - Loss of engagement over time due to internal tensions and/or to interest |

*Attempted to limit the factors of retention and attrition to those measures taken outside of what was offered by the intervention, i.e. actions taken by researchers. However, there are of course some overlap with intervention factors.*

**Table 5.** Strategies that made it easier/better for participants but made it harder/increased limitations for researchers

| **References** | **Group** | **Study/ expectations of participation** | **Challenges for researchers, made it easier for participants** |
| --- | --- | --- | --- |
| **[1]** | Socially disadvantaged women **WHO** have a low household income, have migration backgrounds, are unemployed, rely on welfare aid and/or are a single mother **RECRUITED FROM** an existing community support program for women | **Existing program setting/intervention:** BIG project was established at several sites and promoted physical activity amongst socially disadvantaged women by offering classes and social activities with other members  **Study:** Interview study after 1+years of participation in the program | **Inaccurate interpreters’ interpretation of interviews:** women were allowed to choose who acted as an interpreter, including family members, who may have only translated the meaning of the conversation not the verbatim version which may have resulted in data being lost or slightly changed |
| [2] | marginalized groups of men **WHO** have been regarded as being more difficult to engage with conventional health services, and are **RECRUTIED** **FROM** an existing community support program for men | **Existing program setting:** Sheds are autonomous grass roots spaces which are non-structured and informal, varying in size and resources to enhance the health and wellbeing of the men who attend with social support, in particular (frequently reported as a key enabler of men’s help-seeking). non-clinical nature of the community setting is recognized as a key enabler of men’s engagement in health promotion programs.  **Intervention:** “Shed’s For Life” is a ten-week initiative that delivers targeted and tailored health promotion directly in the Sheds setting.  **Study:** data collection over -3, 0,3 (following 10wk intervention),6,12months | **Capacity constraints of data collectors vs. Sheds different operational types negatively affected follow up rates:** Due to capacity constraints at the time of data collection in Sheds—namely, the availability of two/three data collectors to cover all Sheds and counties as well as the requirement of having to align data collection with Shedder availability and limited Shed opening hours—there were some limitations in terms of the control group and follow-up rates where rescheduling of data collection was not possible.  **Informality/sporadic attendance at Shed venues negatively affected follow up:** Setting: in keeping with the gender specific approach of SFL, Conducting intervention and follow up at Shed venues was challenging for control group (follow up rates where rescheduling of data collection was not possible) considering the informality and sporadic attendance in Sheds.  **Respecting autonomy:** respecting the autonomy of Shedders to opt in/out of the program on their terms took precedence over any attempts to generate a larger size control group.  **Recruitment approach is appropriate but not generalizable:** while the evidence suggests that the recruitment strategy was effective in engaging the target group of shedders, this approach may lead to a potential selection bias when applied to hard to reach groups outside of sheds.  **Sheds setting made randomized study design unfeasible:** due to capricious informal environment, with this complex real world system that has many evolving variable.  **Unbalanced study design to ensure all participants received intervention:** The researcher has to consider an unbalanced design because of the small sample size for control group, which was waitlisted and received the intervention after 3 months (control group data for only 3months). Research has demonstrated that there is value in having a small cohort with a larger intervention group in community based programs where there are often capacity constraints  **Respecting autonomy by offering shedders to opt in/out:** of the program on their terms which took precedence over any attempts to generate a larger size control group. |
| [3] | Men's Sheds members **WHO** are older, likely retired/unemployed, from low socio-econ backgrounds, lower education and health literacy and whose avoidance of health promotion and services is rooted in traditional masculinity such as self-reliance and competitiveness, and whom health services are not adequately tailored to (global gender equity policy), and are **RECRUTIED** **FROM** an existing community support program for men  **Same study as Mcgrath, A., et al. [2]* | **Intervention:** This study captures a baseline cross-sectional analysis of Shedders (n = 384) who participated in ‘Sheds for Life’, a health promotion initiative tailored to Sheds. | **Drop out measures not accurate due to informal nature of the Sheds:** absence of data for a participant does will not necessarily indicate dropout from SFL  **Non-representative sample:** For pragmatic reasons, the sample was drawn from selected counties in Ireland, therefore findings cannot purport to be representative of all Irish Shedders, some of whom are part of underrepresented populations and who may feel stigmatized or labelled in this setting. |
| [8] | women migrants in a Swedish context **WHO** are from socially deprived areas (i.e. low socioeconomic status and widespread criminality that affects the local society, living with stressors such as housing or work, and are **RECRUITED FROM** an existing co-creative lab focusing on women’s health | **Existing program setting:** A co-creative lab/program initiated by women migrants. Activities mainly focusing on social inclusion, started for women within the community.  **Intervention:** This study was part of the program Collaborative Innovations for Health Promotion (CBPR approach). three story dialogues were conducted with 5–8 women in each group. Before each dialogue, one participant in each group prepared a story on the topic of a health issue encountered in daily life. Lay Health Promotors (LHP) facilitated recruitment. | **Lack of representative sample:** study sample was determined by the community interviews. There is a risk that this method of sampling doesn’t generate a representative sample, although this is a method suitable in hard-to-reach populations and was therefore chosen to be used in this study.  **Data collection time limited by participants’ time constraints:** However, in this study only one story per group was included, due to time limitation among the participants.  **Work load of community partners:** To have more personal contact besides general messages is inevitably demanding for the health promoter, while creating a safe atmosphere for the participants. |
| [9] | Women migrants in a Swedish context from the co-creative lab **WHO** also experienced adverse experiences during transit and post-migration (e.g. social isolation, unequal distribution of power and resources), discrimination, with lower mental quality of life, low trust between residents, and have difficulties engaging in physical activity, and are **RECRUITED FROM** a co-creative lab focusing on women’s health  **Same study as Lindsjö, C., et al. [8]* | *(same as above)* | **Recruitment based on LHP’s capacity may have led to missing potential participants:** the inclusion of women happened based on the LHP’s outreaching capacity. First, the LHP used her social network to invite women, and then she invited women that she happened to come across during everyday life, others may have been excluded and there is no control of what persons are excluded.  **Loyalty to LHP may have skewed reality (more positive than reality) of participant responses:** the LHP who facilitated the health-promotive activities was also the facilitator of the story dialogs, where accounts of the women’s experiences of the activities were collected. Very few negative accounts were shared, which could possibly be due to loyalty to the LHP. |
| **[5]** | Socially disadvantaged young to middle-aged disadvantaged men **WHO** are socially disadvantaged with low income, whom uptake of public health interventions is low, and not tailored to them, and are **RECRUTIED FROM** socially disadvantaged areas who had two or more episodes of binge drinking (> 8 UK units on a single occasion) in the preceding 28 days | **Intervention:** All participants received a series of interactive text messages during a period of 12 weeks. The intervention group was sent 112 messages with up to four messages sent on a single day, emphasizing the importance of motivational and volitional phases of the change process, and incorporates behavioral intentions, planning, action and maintenance of the new behavior. The control group received 89 texts on general health, with no mention of alcohol or use of behavior change techniques. | **Incompatibility between strategies for minimizing impact of data collection methods on outcomes and increasing retention:** A possible weakness of study design , i.e. use of an active or attentional control, comprising text messages of trivia on a variety of health topics. intended to increase retention in the study, but could also have influenced drinking behavior by prompting the men to think about their health and their drinking. There is clearly a tension between minimizing the effect of the control package and maximizing retention. The issue is whether it is better to prevent differential loss to follow-up or to reduce research participation effects. However, the bias resulting from loss to follow-up would threaten the validity of the trial, making it the more serious concern. |
| **[20]*** | Moluccans **WHO** are living in Moluccan districts in Netherlands with a history of mistreatment and distrust and culture of individualism and privacy **RECRUITED VIA** direct recruitment door to door and announcement letter.  **ARTICLE PRESENED** primary analysis of recruitment strategies and secondarily, low budget surveys | **Intervention:** Low budget health surveys | “The total fieldwork period was limited to about 8 months, during which the address lists had to be created, and recruitment strategies had to be developed and implemented in 19 MDs throughout the Netherlands. The recruitment period per MD varied from 3 to 7 months. Due to limited financial resources, we could not apply resource-demanding strategies that are potentially effective, such as telephone follow-up and substantial financial rewards to all respondents. Finally, for reasons related to their unique history, Moluccans cannot be identified as such in the Dutch population registry, and we had to invest considerable time in creating address lists that included all eligible persons.” |
| **[22]*** | Residents from disadvantaged neighborhoods, adults **WHO** have mental, social or physical issues **RECRUITED VIA** Face to face recruitment by health professionals (HPs) for intervention.  **ARTICLE PRESENTED** recruitment strategies | **Intervention:** increase interest in health promotion and disease prevention services  **Study:** Door-to-door recruitment by HPs | **Active recruitment:** active recruitment approaches are time intensive and resource demanding  **Challenge of comparing approaches due to individualization of recruitment strategies per HP for each person:** The HPs stated that presenting health services when meeting with residents during active recruitment is difficult because services are extensive and tailored to the resident's individual needs and wishes. One of the key services is ‘health conversations’. How this service was introduced differed among the three HPs. However, Meeting the HPs was experienced as something different. It made residents take in the message, furthered their attention and gave them an experience of meaningfulness and Generally, the residents explained that a HP presenting the services face-to-face increased their interest in the services: |

** Complementary articles that presented relevant information in addition to or instead of outcomes or primary research directly from target group members.*

*NA: not available*

## References

1. Kreiml, V., et al., *“That's like therapy”—A qualitative study on socially disadvantaged women's views on the effects of a community-based participatory research project on their health and health behavior.* Frontiers in Public Health, 2024. **12**: p. 1339556.

2. McGrath, A., et al., *An economic evaluation of ‘Sheds for Life’: a community-based men’s health initiative for men’s sheds in Ireland.* International Journal of Environmental Research and Public Health, 2022. **19**(4): p. 2204.

3. McGrath, A., et al., *Understanding shedders: Which socio‐demographic, health and wellbeing characteristics best inform appropriate health promotion action in men's sheds and a ‘Shed for Life’?* Health Promotion Journal of Australia, 2023. **34**(1): p. 156-168.

4. Irvine, L., et al., *Can text messages reach the parts other process measures cannot reach: an evaluation of a behavior change intervention delivered by mobile phone?* PLoS One, 2012. **7**(12): p. e52621.

5. Crombie, I.K., et al., *Texting to Reduce Alcohol Misuse (TRAM): main findings from a randomized controlled trial of a text message intervention to reduce binge drinking among disadvantaged men.* Addiction, 2018. **113**(9): p. 1609-1618.

6. King, E., et al., *Engaging the agricultural community in the development of mental health interventions: a qualitative research study.* BMC psychiatry, 2023. **23**(1): p. 399.

7. Lock, K., et al., *Evaluating social and behavioural impacts of English smoke-free legislation in different ethnic and age groups: implications for reducing smoking-related health inequalities.* Tobacco control, 2010. **19**(5): p. 391-397.

8. Lindsjö, C., et al., *Health promotion focusing on migrant women through a community based participatory research approach.* BMC Women's Health, 2021. **21**: p. 1-12.

9. Lindsjö, C., et al., *Migrant women’s engagement in health-promotive activities through a women’s health collaboration.* Frontiers in Public Health, 2023. **11**: p. 1106972.

10. Thielecke, J., et al., *How to promote usage of telehealth interventions for farmers' mental health? A qualitative study on supporting and hindering aspects for acceptance and satisfaction with a personalized telephone coaching for depression prevention.* Internet Interventions, 2023. **34**: p. 100671.

11. Helitzer, E., H. Moss, and J. O’Donoghue, *Lifting spirits and building community: the social, emotional and practical benefits of all-female group singing.* Health Promotion International, 2022. **37**(6): p. daac112.

12. McRobbie, H., et al., *Randomised controlled trial and economic evaluation of a task-based weight management group programme.* BMC Public Health, 2019. **19**: p. 1-10.

13. Kolovou, V., et al., *Recruitment and retention of participants from socioeconomically deprived communities: lessons from the Awareness and Beliefs About Cancer (ABACus3) Randomised Controlled Trial.* BMC Medical Research Methodology, 2020. **20**: p. 1-9.

14. Grazioli, V.S., et al., *Six-month outcomes among socially marginalized alcohol and drug users attending a drop-in center allowing alcohol consumption.* International Journal of Drug Policy, 2017. **41**: p. 65-73.

15. D'Hooghe, S., et al., *The role of the perceived environment for recreational walking among adults in socioeconomically disadvantaged situations: A study using walk-along interviews.* SSM-Population Health, 2023. **23**: p. 101456.

16. Milcarz, M., et al., *Tobacco health risk awareness among socially disadvantaged people—A crucial tool for smoking cessation.* International journal of environmental research and public health, 2018. **15**(10): p. 2244.

17. Sinclair, A. and H. Alexander, *Using outreach to involve the hard-to-reach in a health check: what difference does it make?* Public Health, 2012. **126**(2): p. 87-95.

18. Craddock, E., *A qualitative UK study exploring counterpublic engagement of marginalized women via a Women’s Health Network.* Health Promotion International, 2022. **37**(4): p. daac124.

19. Mueller, J., et al., *Feasibility of a community-based cancer awareness initiative: views of those delivering and managing the intervention.* Journal of Public Health, 2023. **31**(5): p. 765-771.

20. Bodewes, A.J. and A.E. Kunst, *Involving hard-to-reach ethnic minorities in low-budget health research: lessons from a health survey among Moluccans in the Netherlands.* BMC research notes, 2016. **9**: p. 1-8.

21. Ridley, J., S. Hunter, and A. Rosengard, *Partners in care?: views and experiences of carers from a cohort study of the early implementation of the Mental Health (Care & Treatment)(Scotland) Act 2003.* Health & Social Care in the Community, 2010. **18**(5): p. 474-482.

22. Bysted, S., et al., *Recruiting residents from disadvantaged neighbourhoods for community‐based health promotion and disease prevention services in Denmark—How, why and under what circumstances does an active door‐to‐door recruitment approach work?* Health & Social Care in the Community, 2022. **30**(3): p. 937-948.

23. Lewis, S., et al., *Reframing “participation” and “inclusion” in public health policy and practice to address health inequalities: Evidence from a major resident‐led neighbourhood improvement initiative.* Health & social care in the community, 2019. **27**(1): p. 199-206.

24. Sjögren Forss, K., A. Kottorp, and M. Rämgård, *Collaborating in a penta-helix structure within a community based participatory research programme:‘Wrestling with hierarchies and getting caught in isolated downpipes’.* Archives of Public Health, 2021. **79**: p. 1-13.
